# Role in Diuresis of a Calcitonin Receptor (GPRCAL1) Expressed in a Distal-Proximal Gradient in Renal Organs of the Mosquito *Aedes aegypti* (L.)

**DOI:** 10.1371/journal.pone.0050374

**Published:** 2012-11-29

**Authors:** Hyeogsun Kwon, Hsiao-Ling Lu, Michael T. Longnecker, Patricia V. Pietrantonio

**Affiliations:** 1 Department of Entomology, Texas A&M University, College Station, Texas, United States of America; 2 Department of Statistics, Texas A&M University, College Station, Texas, United States of America; New Mexico State University, United States of America

## Abstract

Evolution of anthropophilic hematophagy in insects resulted in the coordination of various physiological processes for survival. In female mosquitoes, a large blood meal provides proteins for egg production and as a trade-off, rapid elimination of the excess water and solutes (Na^+^, Cl^−^) is critical for maintaining homeostasis and removing excess weight to resume flight and avoid predation. This post-prandial excretion is achieved by the concerted action of multiple hormones. Diuresis and natriuresis elicited by the calcitonin-like diuretic hormone 31 (DH_31_) are believed to be mediated by a yet uncharacterized calcitonin receptor (GPRCAL) in the mosquito Malpighian tubules (MTs), the renal organs. To contribute knowledge on endocrinology of mosquito diuresis we cloned GPRCAL1 from MT cDNA. This receptor is the ortholog of the DH_31_ receptor from *Drosophila melanogaster* that is expressed in principal cells of the fruit fly MT. Immunofluorescence similarly showed *Aaeg*GPRCAL1 is present in MT principal cells in *A*. *aegypti*, however, exhibiting an overall gradient-like pattern along the tubule novel for a GPCR in insects. Variegated, cell-specific receptor expression revealed a subpopulation of otherwise phenotypically similar principal cells. To investigate the receptor contribution to fluid elimination, RNAi was followed by urine measurement assays. *In vitro*, MTs from females that underwent *AaegGPRcal1* knock-down exhibited up to 57% decrease in the rate of fluid secretion in response to DH_31_. Live females treated with *AaegGPRcal1* dsRNA exhibited 30% reduction in fluid excreted after a blood meal. The RNAi-induced phenotype demonstrates the critical contribution of this single secretin-like family B GPCR to fluid excretion in invertebrates and highlights its relevance for the blood feeding adaptation. Our results with the mosquito *Aaeg*GPRCAL1 imply that the regulatory function of calcitonin-like receptors for ion and fluid transport in renal organs arose early in evolution.

## Introduction

Insect diuresis is regulated by the concerted action of different hormones, among them, peptide hormones such as calcitonin-like diuretic hormone 31 (DH_31_), corticotropin-releasing factor (CRF)-like diuretic hormone 44 (DH_44_), insect kinins, CAPA-periviscerokinins, arginine vasopressin-like insect diuretic hormones and serotonin [1]–[4]. These act on cognate receptors in the renal organs, the Malpighian tubules (MTs), to stimulate ion transport for primary urine production and subsequent fluid excretion. In hematophagous female mosquitoes excess fluid and ions (Na^+^, Cl^−^, K^+^) acquired with the blood meal are transported from the hemolymph to the MT lumen to secrete urine at extremely high rate [5], [6]. Transport is achieved by channels and co-transporters in the MT principal and stellate cells and through septate junctions between principal cells and is energized by an apical vacuolar H^+^-ATPase (V-ATPase) in the principal cells. The latter have the fastest ion transporting capacity of any cell so far studied, resulting in high rates of fluid secretion [7]–[10]. The immediate fluid excretion from females that eliminates excess weight is believed to have contributed to the successful adaptation to blood feeding in mosquitoes, at minimum by avoiding predation [11].

A calcitonin-like peptide (diuretic hormone 31; DH_31_) named “mosquito natriuretic factor” [12] previously to its molecular identification [3], [13], induces fluid secretion in the MTs and is the main hormone stimulating natriuresis post-blood feeding [14]. Only one gene for DH_31_ is predicted in the genome of the mosquito *Aedes aegypti* (GenBank: XM_001658818) and those of the fruit fly *Drosophila melanogaster* (CG13094) and other insects; correspondingly only one such peptide has been isolated in *A. aegypti* [3].

In vertebrates, two hormones, calcitonin (CT) and calcitonin gene related peptide (CGRP), have comparable activities to DH_31_ peptide in maintaining renal homeostasis by increasing water, electrolytes, and urea excretion [15]–[17]. CT signals through the calcitonin receptor (CALCR) and CGRP through calcitonin receptor-like receptor (CALCRL) and both GPCRs are expressed in the kidney and other organs [18], [19]. Similar to the mammalian CALCRL receptor [20], the recombinant *D. melanogaster* calcitonin receptor-like receptor 1 (GPRCAL1) required mammalian receptor-activity-modifying proteins (RAMPs) for activity though no RAMP orthologues are found in known Dipteran genomes [21]. In insects a number of studies have demonstrated that DH_31_ peptides stimulate diuresis [13], [22], [23]; however, less is known about the function of their cognate receptors *in vivo*. The volume of fluid secreted by MTs or excreted *in vivo* by individual activation of the DH_31_ receptor is unknown in any insect.

To contribute knowledge on the endocrine regulation of MTs in female mosquitoes, we cloned the ortholog of *Drosophila* DH_31_ receptor, *Aaeg*GPRCAL1, and investigated the receptor role in diuresis through a multifaceted approach. We found that *Aaeg*GPRCAL1 is expressed in principal cells in a distal-proximal gradient in the MTs, novel for an insect GPCR and revealing principal cell specialization or receptor regulation. This spatial distribution further uncovers a signaling mechanism that establishes a longitudinal osmotic gradient to drive fast fluid secretion in MTs. Knockdown of *AaegGPRcal1* caused a significant reduction in MTs primary urine secretion and excretion in live females. Our results indicate that signal transduction through this evolutionarily ancient GPCR significantly regulates diuresis after a blood meal, perhaps contributing to the success of the blood feeding adaptation in mosquitoes.

## Materials and Methods

### Mosquito Colony Maintenance

*A*. *aegypti* (Diptera; Culicidae), Rockefeller strain, was reared as described [24]; blood feeding was done on membrane feeders. Only females were used for all experiments.

### mRNA Isolation and Cloning of *AaegGPRcal1*

A NCBI BLASTP search of *A*. *aegypti* genomic shotgun sequences was performed with the GPRCAL1 (CG32843) *Drosophila* sequence [21] and conserved protein regions for *Aaeg* GPRCAL1 were identified in contig GenBank AAGE02017873. Specific primers were designed (Table S1) based on this sequence to amplify the full length cDNA of *AaegGPRcal1.*

To obtaine cDNA, MTs from 3 to 5-day-old non-blood fed females were dissected and the Dynabeads® mRNA Direct Kit (Invitrogen, Grand Island, NY, USA) was used for isolation of mRNAs from ∼170 MTs. cDNA was synthesized with the RLM-RACE kit (GeneRacer™, Invitrogen). Designed primers were used to amplify the receptor cDNA with the Advantage® 2 PCR kit (Clontech, Mountain View, CA, USA). After gel electrophoresis the PCR product was extracted with QIAquick kit (Qiagen, Valencia, CA, USA) and cloned into the pCR®2.1-TOPO®vector with the TOPO TA cloning kit (Invitrogen). Plasmid DNA was purified with a Qiaprep spin miniprep kit (Qiagen) and sequenced using ABI PRISM Big Dye Terminator Cycle sequencing Core kit (Applied Biosystems, Carlsbad, CA, USA).

### Western Blotting

Membranes from MTs were prepared as described [25]. MTs (∼2500) were dissected from 3 to 5- day-old non-blood fed females and homogenized in cold buffer (25 mM Tris-HCl at pH 7.5, 1 mM EDTA, 1 mM EGTA, 1 mM dithiothreitol) with a protease inhibitor cocktail (Roche, Indianapolis, IN, USA). The homogenates were centrifuged at 800×g for 5 min and the supernatants were collected. The remaining pellets were re-homogenized and centrifuged again. All supernatants were centrifuged at 100,000×g for 1 h at 4°C. After ultracentrifugation, the pellets were re-dissolved in 200 µl cold buffer (50 mM Tris-HCl, pH 7.5, 2 mM CaCl_2_) with protease inhibitors, and stored at −80°C until used for western blot analysis. Protein concentration of membrane preparations was measured with the BCA assay kit (Pierce, Rockford, IL, USA) and the proteins were treated with PNGase F (New England Biolabs, Ipswich, MA, USA) in a glycoprotein denaturing buffer with 10% NP 40. Samples were incubated at 37°C for 1 h. The proteins were added 5× sample buffer (125 mM Tris at pH 6.8, 4% SDS, 20% glycerol, 0.004%, dithiothreitol, and 1% bromophenol blue) and boiled for 5 min. Proteins from ∼500 MTs (80 µg) per lane were separated on 10% SDS/PAGE gel (Bio-Rad, Hercules, CA, USA) in Tris–glycine electrophoresis buffer (25 mM Tris, 250 mM glycine, and 0.1% SDS) for 75 min at 120 V. Proteins were transferred to PVDF (poly-vinylidene difluoride) membranes (Millipore, Billerica, MA, USA) that were blocked in TBST buffer (10 mM Tris base, 140 mM NaCl, 0.1% Tween, pH 7.4) containing 5% non-fat milk for 1 h at room temperature. The membrane was incubated overnight in blocking buffer with 1∶500 diluted rabbit anti-*Aaeg*GPRCAL1 (C-RGYAGTPEDTIE: second extracellular loop) affinity purified antibodies (0.5 mg/ml) (Pacific Immunology, Ramona, CA, USA) on a shaker overnight at 4°C. After washed 3×10 min in TBST, the membrane was incubated with 1: 40,000 horseradish peroxidase-conjugated goat anti-rabbit IgG (Jackson Immunoresearch, West Grove, PA, USA) for 1 h at room temperature. Following 3×10 min in TBST washes, the membrane was incubated for 5 min in the dark with 1 ml of SuperSignal®West Pico Chemiluminescent Substrate (Thermo Scientific, Rockford, IL, USA). Anti-*Aaeg*GPRCAL1 affinity purified antibody (1∶500 dilution; 1 µg/ml) pre-absorbed overnight at 4°C with *Aaeg*GPRCAL1 peptide antigen (100 µg in 5% non-fat milk in TBST) was used as a negative control (∼100×molar excess).

### Whole Mount Immunofluorescence and Statistical Analyses

Analyses were performed as described [26]. The MTs from individual 6 to 8-day-old non-blood fed females were dissected attached to the pylorous-midgut junction and fixed for 4 h in 4% paraformaldehyde (Sigma-Aldrich, St Louis, MO, USA) in PBS at 4°C on a shaker. After 3×10 min washes with 70% ethanol, the tissues were washed again with PBST (0.1% Tween) 2×10 min. Tissues were treated with proteinase K (12 ng/µl) for 5 min and rinsed twice for 5 min with PBST. Tissues were treated with image-iT™ FX signal enhancer (Invitrogen) for 30 min at room temperature and then blocked in 10% normal goat serum (NGS) in PBST overnight at 4°C. Tissues were incubated 24 h at 4°C with affinity purified anti-*Aaeg*GPRCAL1 antibodies (1∶100 and 1∶250) for receptor localization, and pre-immune serum (1∶2000) and pre-absorbed anti-*Aaeg*GPRCAL1 affinity-purified antibodies (1∶250) for negative controls, respectively. Pre-absorbed antibodies were prepared by incubation in the anti-*Aaeg*GPRCAL1 antibody (6 µl) with the *Aaeg*GPRCAL1 antigen (100 µg) in PBSTG (2% normal goat serum) overnight at 4°C. After 4×20 min wash in PBSTG, tissues were incubated with Alexa 555 goat anti-rabbit highly cross-adsorbed IgG (1∶200; Invitrogen) in PBSTG for overnight at 4°C. Tissues were washed for 6×30 min in PBSTG. Individual female MTs could be identified for analyses because they remained attached to the pylorus. Tissues were mounted in Vectashield®medium with 4′, 6-diamidino-2-phenylindole (DAPI) for nuclear staining (Vector, Burlingame, CA, USA) and observed under a Carl Zeiss Axioimager A1 microscope with filters for DAPI (G 365 nm, FT 395 nm, BP 445 nm) and Alexa Fluor 555 (BP 546 nm, FT 560 nm, BP 575–640 nm). Images were obtained with an AxioCam MRc color camera and analyzed with Axiovision software (Carl Zeiss Microimaging, Thornwood, NY, USA). Confocal images were obtained with an Olympus FV1000 confocal microscope equipped with a 20X/0.85 and 100X/1.4 oil immersion objectives and 405 nm and 543 nm lasers for excitation. Sequential scanning was used to minimize fluorescence channel cross-talk. The images were analyzed with FV10-ASW 1.6 Viewer (Olympus America Inc, Center Valley, PA, USA) at the microscopy and imaging center, Texas A&M University.

The probability of *Aaeg*GPRCAL1 immunofluorescence signal along the length of the MT (considered in this study as composed of 54 principal cells), was analyzed from 42 non-blood fed females, as follows. A generalized linear mixed model (GLMM) PROC GLIMMIX (SAS version 9.2, SAS Institute Inc., Cary, NC, USA), was used first to determine whether there were significant differences in the probability of principal cells exhibiting receptor signal depending on the principal cell position along the tubule. “Presence of signal in principal cell” and the “individual mosquitoes” were considered as nominal variable and a random effect, respectively. Second, a logistic regression model was used to quantify the relationship between the probability of receptor signal and position of principal cells. In this model, “mosquito” was still considered as a random effect but “principal cell position” was considered a fixed factor and a continuous variable with values 1 to 54. This produced the following equation for estimating the probability (P) that a cell exhibits receptor signal based on its position (Pos.), with the tip cell being in position #1 and the last proximal cell in position #54. 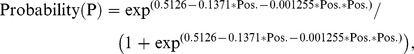
where Pos. is a value between 1 and 54. The plot of signal probability versus principal cell position was created by this equation.

To verify the RNAi effect on receptor protein expression, MTs were dissected from females 7 days post-injection. For immunohistochemistry, anti-*Aaeg*GPRCAL1 antibodies were used 1∶250. Images were quantitatively analyzed by calculating the difference in maximal pixel signal intensity of control tissues vs. those of females injected with *AaegGPRcal1* dsRNA using the Image-Pro Plus (Media Cybernetics, Acton, MA, USA) software.

### RNAi and RNAi Evaluation by qPCR and Fluid Secretion Assays

1. dsRNA synthesis and microinjection. The N-terminus of *AaegGPRcal1*, containing the 5′ UTR and the coding region encompassing the first 85 amino acid residues was chosen for dsRNA synthesis; genome searches did not identify any similar regions, thus minimizing the probability of non-target effects. Primers specific for *AaegGPRcal1* and enhanced green fluorescent protein (EGFP) flanked with the T7 promoter sequence were designed (Table S1). The pCR®2.1-TOPO plasmid containing the *AaegGPRcal1* cDNA was used to amplify a 349 bp product; the latter and a 612 bp product from EGFP (GenBank: U55763.1; Lit 28i polylinker EGFP) were used as the templates for dsRNA synthesis. MEGAscript RNAi kit (Ambion, Austin, TX, USA) was used to synthesize dsRNA following the manufacturer’s instructions. RNA was precipitated with ammonium acetate-ethanol and centrifuged (15 min) at 4°C at 13628×g; after dried in air, the pellet was dissolved in nuclease free water. Injections were with Femtotip® needles (Eppendorf, Hamburg, Germany) connected to a FemtoJet® microinjector (Eppendorf). For all RNAi experiments one-day-old, non-blood fed females were anesthetized on ice and injected in the thorax with ∼1.2–1.5 µg *AaegGPRcal1* dsRNA, ∼1 µg EGFP dsRNA or ∼150 nl water; the last two treatments served as negative controls. The injected females were allowed to recover for one day before males were introduced to mate. Mosquitoes were kept at 27°C (16L:8D) fed 10% glucose-water and starved for 24 h prior to blood feeding.
2. RNAi and evaluation by qPCR. The optimum time period(s) post-injection used as end points for RNAi evaluation were based on the results of RNAi pilot experiments. In these, females were analyzed by qPCR (at 5 and 7 days post-injection) and by fluid excretion in a precision humidity chamber (from 5–11 days post injection) (see subsection 4). The pilot experiments determined that qPCR was best evaluated 5 days post-injection and fluid excretion 7 days post-injection (see subsection 4). For RNAi females were injected with *AaegGPRcal1* dsRNA, EGFP dsRNA, or water (*N* = 60−70 for each treatment per replicate; three independent replications were performed). For qPCR evaluation the MTs from females in each replicate were dissected 5 days post-injection into RNAlater®tissue collection solution (Ambion) and mRNA was isolated with Dynabeads®mRNA Direct kit. Single strand cDNA was synthesized for each replicate with SuperScript™III reverse transcriptase (200 U/µl; Invitrogen). For qPCR, SYBR®Green PCR Master Mix (Applied Biosystem) was prepared for each cDNA template as follows: 60 µl SYBR green reagent was added to 6 µl cDNA template (∼42–57 MT equivalent) and 10.8 µl of water. The total volume of 76.8 µl was equally divided for analyses of the *AaegGPRcal1* and β-actin transcripts, respectively. Either amplicon primers (5 µM each in 10.8 µl) for *AaegGPRcal1* (AADH31FQPCR3’ORF and AADH31RQPCR3’ORF) or β-actin (P178 and P179) were added for a final concentration of 900 nM in the reaction (Table S1). This amplification was performed using an ABI 7300 (Applied Biosystem).
3. Fluid secretion assay from an individual MT. Fluid secretion from individual MTs was measured by Ramsay assay [27]. Seven days post-injection, MTs from females from the three treatments (*AaegGPRcal1* dsRNA, EGFP dsRNA or water) were carefully isolated in Ringer saline (150 mmol l^−1^ NaCl, 25 mmol l^−1^ Hepes, 3.4 mmol l^−1^ KCl, 7.5 mmol l^−1^ NaOH, 1.8 mmol l^−1^ NaHCO_3_, 1 mmol l^−1^ MgSO_4_, 1.7 mmol l^−1^ CaCl_2_ and 5 mmol l^−1^ glucose, pH 7.1) [28]. Forceps and pipette tips were coated with 1% bovine serum albumin, and rinsed with saline to prevent tissue from sticking. The MTs in 30 µl drop were transferred to 20 ml light paraffin oil bath. The proximal one third of the MT was pulled from the saline into the oil by glass hooks holding the open distal end. The diameter of the secreted droplet was measured with an ocular reticle (VWR, West Chester, PA, USA) mounted in the dissecting microscope (Olympus SZ60). To establish the baseline excretion, measurements were taken every 5 min after 10 min of equilibration. After this control period secretion rate was measured, *Aedes* diuretic hormone 31 (*Aaeg*-DH_31_) was applied (2 µmol l^−1^ in 30 µl) into the saline reservoir, and 30 µl of saline was removed to maintain the same volume. Upon hormone application the secreted droplet was measured at 5, 10, 15, 20 and 40 min. The secreted volume was calculated from the dimensions of a spheroid and was calculated using the formula (V = (πa^2^b)/6×10^6^ were (b) is the long and (a) is the short diameter). An *A*. *gambiae* 95 amino acid precursor peptide (accession number: XM_321755) 84% identical to *Drome*-DH_31_ was used to identify *Aaeg*-DH_31_ through a genome protein blast search. *A*. *aegypti* DH_31_ (accession number EAT40182) was synthesized (GenScript Corporation, Piscataway, NJ, USA). This *Aaeg*-DH_31_ peptide (TVDFGLSRGYSGAQEAKHRM AMAVANFAGGPa) was recently identified by mass spectrometry from brain tissues [3].
4. Blood feeding and humidity chamber assay. Parafilm was stretched over a glass feeder using a water-jacket to warm defibrinated rabbit blood (Hemostat Laboratories, Dixon, CA, USA), containing 1.67 mg/ml ATP (Sigma-Aldrich) [29]. The parafilm was treated with vaseline. Seven days post-injection female mosquitoes were singly blood fed by keeping them contained over a well containing blood. Females were fed until they removed the stylets, and then were individually assessed in a humidity chamber. Continuing excretory water loss from individual females was measured for 1 h by “Expedata” data acquisition software using a sable system and humidity analyzer (RH-300) (Sable Systems International, Las Vegas, NV, USA). Constant dry air was passed through the chamber under conditions of 1% relative humidity (RH), flow rate 100 ml/min, and temperature between 24–26°C using a Subsampler 3 (Sable Systems International) [30]. The system was calibrated with known volumes of distilled water (0.5 to 2 µl).

### Statistical Analyses

q-RT-PCR: One-way ANOVA followed by Tukey’s test (SPSS, IBM Corporation, Armonk, NY, USA) was used to determine the relative *AaegGPRcal1* abundance in MTs. Three independent experiments were performed with 60–70 injected females per treatment per experiment; a total of about 540 females. RNAi evaluation through *in vitro* and *in vivo* fluid secretion/excretion assays: To determine the effect of treatments through time (1 h), and to measure the interaction between treatments and time at which secretion/excretion measurements were taken, the repeated measures ANOVA was conducted using PROC GLMM followed by the Tukey-Kramer test (SAS).

## Results

### *Aaeg*GPRCAL1 Phylogenetic Analyses and Ligand Structural Conservation

Until present, there has not been a thorough analysis of insect GPRCAL1 receptors [4], or the structural 3-D features of their ligands in insects. A 1995 bp cDNA (GenBank: JQ045343) encoding a 412 amino acid residue receptor protein (46.9 kDa mass) was cloned from female MTs (Fig. S1). Topology prediction and motif scanning analyses confirmed this sequence corresponded to a secretin family GPCR (Fig. S1) [31], [32]. The *AaegGPRcal1* predicted genomic sequence (NCBI contig AAGE02017873) was identical to the obtained cDNA at the 5′UTR and most of the ORF, but was incomplete at the 3′ end, which was located in contig AAGE02019029 (NCBI). *AaegGPRcal1* is the ortholog of the *D. melanogaster* calcitonin receptor-like receptor 1 (*DmelGPRcal1*) (64% amino acid sequence identity) that is activated by *Dmel*-DH_31_ (76% amino acid sequence identity to *Aaeg*-DH_31_) [21].

Phylogenetic analyses based on sequence alignments (Fig. S2) showed that in the secretin GPCR group, corticotropin releasing hormone 1 (CRHR1) receptors and the CALCRLs/CALCRs group belong to two independent clusters, each including receptors from protostomes (mollusks) and deuterostomes (fish, bird, amphibian, and mammals) (Fig. S3). These results suggest that these two receptor groups diverged before the split of insects and vertebrates. Within the CALCRLs/CALCRs group, insect receptors formed one subcluster (GPRCAL1) and appeared distinct from both CALCRLs and CALCRs of protostomes and deuterostomes. The *Aaeg*GPRCAL1 sequence is more identical to human CALCRL (hCALCRL) (33%) than to hCALCR (30%) (Fig. S2). The three conserved Asn glycosylation sites Asn26, 93, 98 (Fig. S1) could be differentially glycosylated as in hCALCRL [33], in which this differential degree of glycosylation affects ligand binding and receptor cell surface expression. Conservation of functionally significant structural similarities between insects and deuterostomes is also likely for their receptor ligands. We then obtained the predicted tertiary structure of the *Aaeg*-DH_31_ based upon the CT Protein Data Base templates from human (PDB ID: 2JXZ) and salmon (PDB ID:2GLH). They all share an α-helix structure, although *Aaeg*-DH_31_ is only ≤16% identical to human CT (or CGRP) (Fig. S4A). While the α-helix of human CGRP is also similar to that of *Aaeg*-DH_31_, homologous key residues for receptor activation were not found in *Aaeg*-DH_31_ (Fig. S4B) [34]. In contrast, the *Aaeg*-DH_31_ C-terminal amidated proline (Fig. S4C) that is also present and critical for hCT activity may have similar receptor-activating function in insects [35]. In summary, *Aaeg*-DH_31_ contains functional features of hCT for receptor activation, but RAMPs may be required for activation of *Aaeg*GPRCAL1, as they are for hCALCRL activation. Mammalian CT exclusively binds CALCR and does not require RAMPs for receptor activation [36].

### *Aaeg*GPRCAL1 Expression and Localization in the MTs

**Figure 1:**
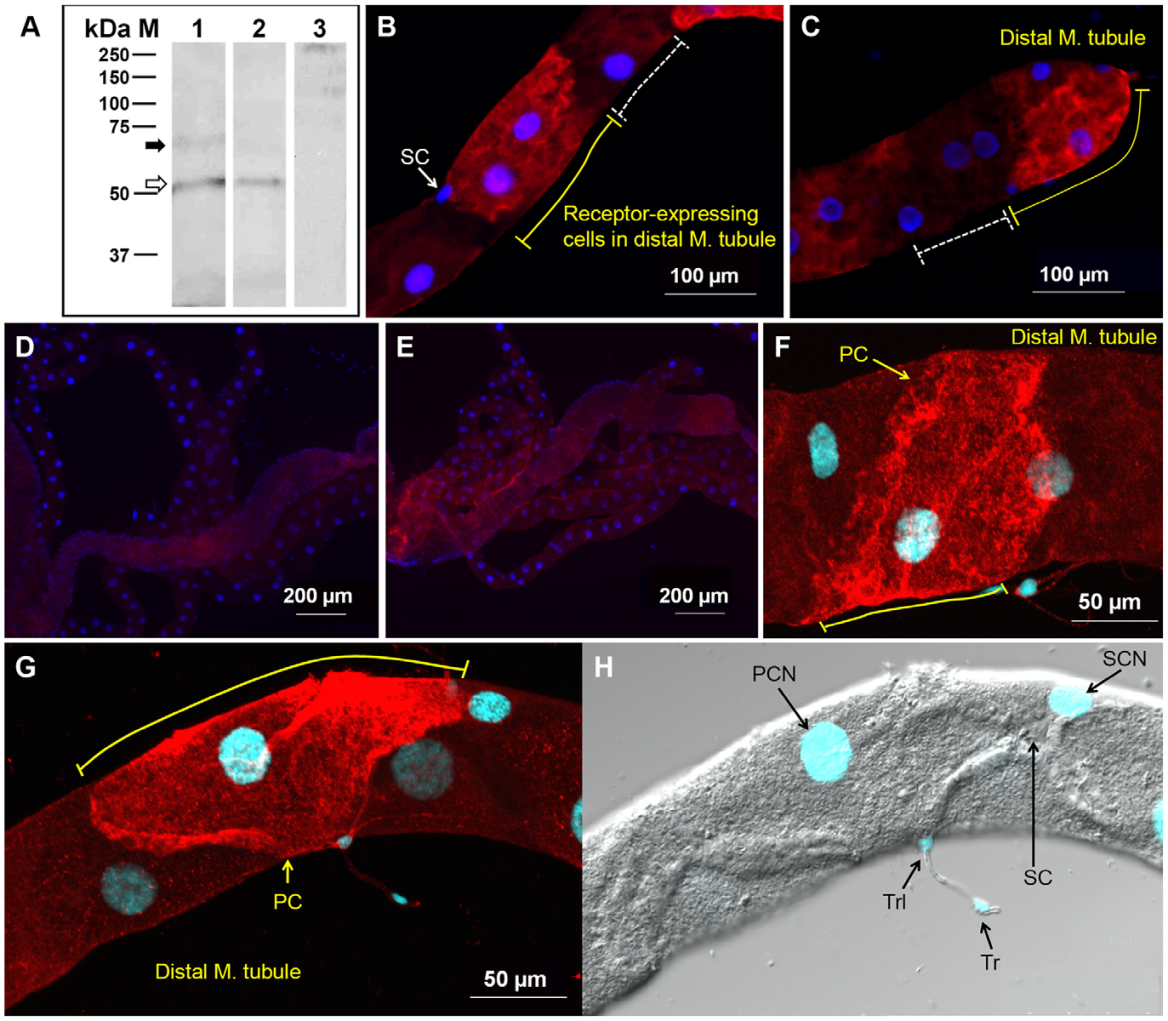
*Aaeg*GPRCAL1 is glycosylated and expressed in selected principal cells in the Mts. (**A**) Western blot of female MT membrane preparations without (lanes 1 and 3) or with (lane 2) PNGase F preincubation and probed with anti-*Aaeg*GPRCAL1 antibody (lanes 1 and 2) or antigen pre-absorbed antibodies (lane 3). Bands at (73 kDa (black arrow) and (51 kDa (open arrow) represent the N-glycosylated and non-glycosylated receptor populations, respectively. (**B–H**) Immunofluorescence analysis of MT whole mounts. Tissues were probed with anti-*Aaeg*GPRCAL1 receptor antibodies (**B, C, F, G**). The yellow solid lines indicate *Aaeg*GPRCAL1 signal in the plasma membrane of principal cells while dashed white lines (**B, C**) indicate absence of signal in adjacent principal cells. Notice receptor expression in MT tip cell in (**C**). No receptor signal was observed in negative control tissues incubated with either antigen pre-absorbed antibodies (**D**) or pre-immune serum (**E**). Confocal microscopy analyses showed the receptor signal only in certain principal cells (PC) (**F, G**). (**H**) DIC confocal photograph of the same tissue as in (**G**) showing the stellate cell (SC), and nuclei in blue (DAPI) of stellate cell (SCN), principal cell (PCN), tracheolar (Trl) and tracheal cells (Tr).

We analyzed the receptor expression in membrane preparations of female MTs by western blot (Fig. 1A). A faint band at (73 kDa (black arrow) and a strong band at ∼51 kDa (open arrow) were specifically recognized by the anti-*Aaeg*GPRCAL1 antibody (lane 1); the latter band slightly higher than the receptor predicted mass. Therefore, these two bands may represent receptor populations with differential modifications such as N-glycosylation and/or phosphorylation (Fig. S1) [20], [37]. To investigate the first possibility, membrane preparations were treated with PNGase F. Although treatment did not modify the size of the lower band, it reduced the intensity of the ∼73 kDa band (Fig. 1*A*, lane 2) in comparison to untreated preparations (Fig. 1A, lane 1). Membrane preparations (without PNGase treatment) probed with the antigen-preabsorbed antibodies showed no specific labeling, as expected (Fig. 1A, lane 3), confirming antibody specificity.

**Figure 2:**
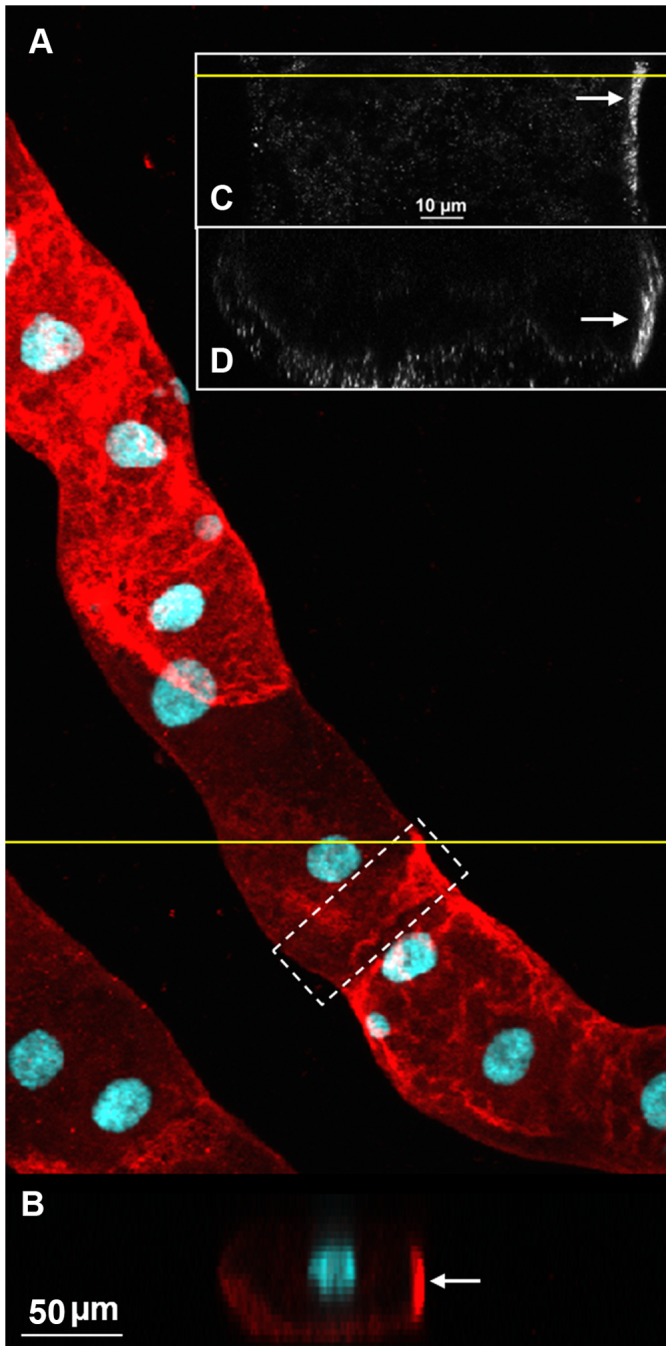
Expression of *Aaeg*GPRCAL1 in the basolateral membrane of principal cell (PC). (**A**) Expression of *Aaeg*GPRCAL1 in principal cells (maximum intensity of projection for a confocal Z-stack; 23 optical sections, Z-step 3.6 µm). (**B**) XZ section of the stack at the position indicated by the yellow line in (**A**). It shows the PC nucleus near the PC apical membrane and receptor signal consistent with the location of the basolateral membrane. A particular section of a PC near the distal tubule (dashed white rectangle in A) was chosen for further analyses in **C** and **D**. (**C–D**) Higher resolution images acquired using a 100X/1.4 oil immersion objective from the white dashed-box area in (**A**). (**C**) A single optical XY section located 10.12 µm from the basolateral membrane. (**D**) XZ view of the Z-stack (86 images, Z-step 0.44 µm) at a position indicated by the yellow line in (**C**). *Aaeg*GPRCAL1 signal in the basolateral membrane region of the PC is indicated with white arrows (**B–D**).

Immunohistochemical analyses of MTs from non blood-fed females with the anti-receptor antibody showed *Aaeg*GPRCAL1 signal in particular principal cells (red, Fig. 1B, C, F, G). Receptor signal was not detected in negative control tissues treated with antigen pre-absorbed antibodies (Fig. 1D) or pre-immune serum (Fig. 1E), as expected. Confocal images revealed that the *Aaeg*GPRCAL1 signal localization is consistent with receptor expression in the basolateral membrane of principal cell (white arrows in Fig. 2 B–D).

**Figure 3:**
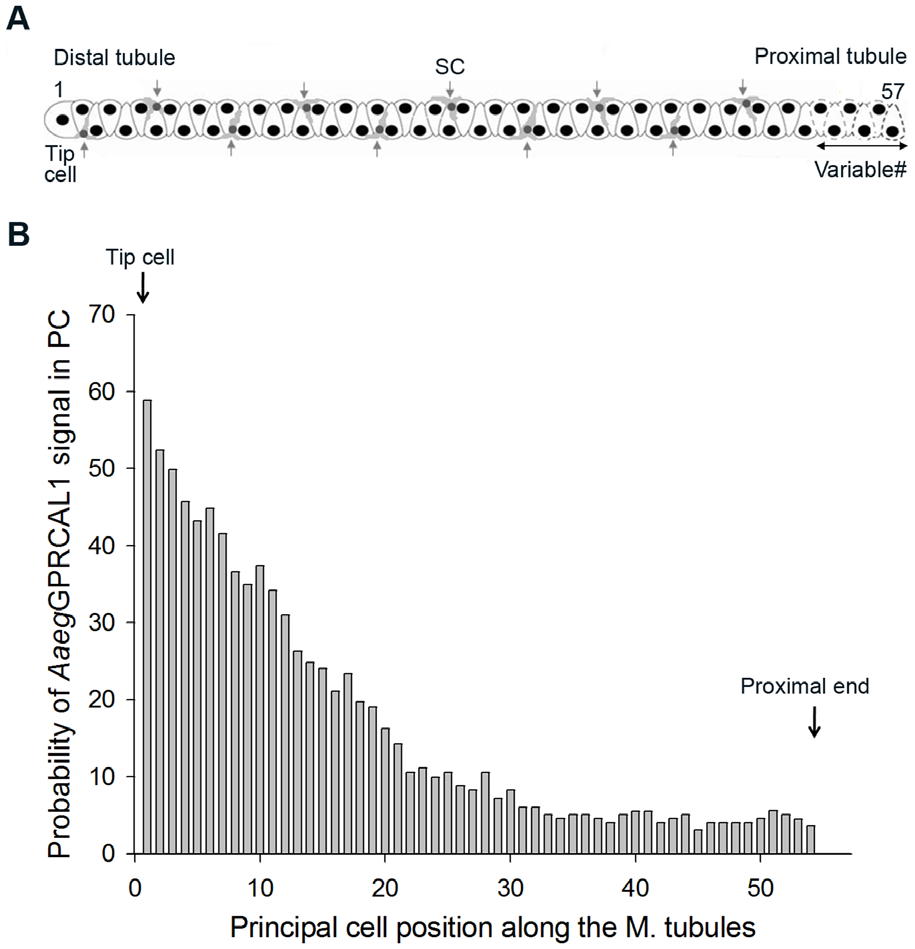
*Aaeg*GPRCAL1 signal in principal cells (PCs) is distributed in a gradient-like fashion along the MTs. (**A**) MT schematic indicating the position of PCs from the tip cell (number 1) to the last proximal cell (variable from number 51 to 57 in individual mosquitoes, dashed cells). The average number of PC (54) and stellate cells (10) per tubule was obtained from MTs analyzed by immunohistochemistry. (**B**) Receptor signal was only observed up to cell number 54 (♀*N* = 42). Plot of probability of receptor signal versus cell position was created by the following equation. Probability (P) = exp ^(0.5126-0.1371*Pos.-0.001255*Pos.*Pos.)^/(1+exp^(0.5126-0.1371*Pos.-0.001255*Pos.*Pos.)^.

*Aaeg*GPRCAL1 signal was not observed in all principal cells even within the same region (Figs. 1B, C, F, G and S5). This observation prompted a quantitative analysis of the probability of principal cells displaying receptor signal in relationship to their position along the length of the MT (Fig. 3A). Statistical analyses confirmed that there was significant difference in the probability of *Aaeg*GPRCAL1 signal across 54 principal cells; the test of a difference in the odds ratio across the 54 cell positions had a *P*-value <0.0001. The random effect of “mosquito” was significant, with an estimated variance of 1.0764 with a S.E. of 0.2573, indicating variability among mosquitoes in the sample studied. Additionally, there was significant evidence of a strong relationship between the probability of *Aaeg*GPRCAL1 signal and the specific position of principal cell along the MT. There is significant evidence (*P*-value ranging from 0.0074 to a value less than 0.0001) that each of the three coefficients in the equation were different from 0 (Fig. 3B). The probability of principal cells expressing *Aaeg*GPRCAL1 signal was higher toward to the distal MTs, indicating the receptor is expressed in a gradient-like fashion along the length of the MTs from the tip cell up to cell position number 29, where the confidence interval of the odds ratio included the value of 1 (Fig. 3B).

### Effects of *Aaeg*GPRCAL1 RNAi in Diuretic Function of *A. aegypti*

**Figure 4:**
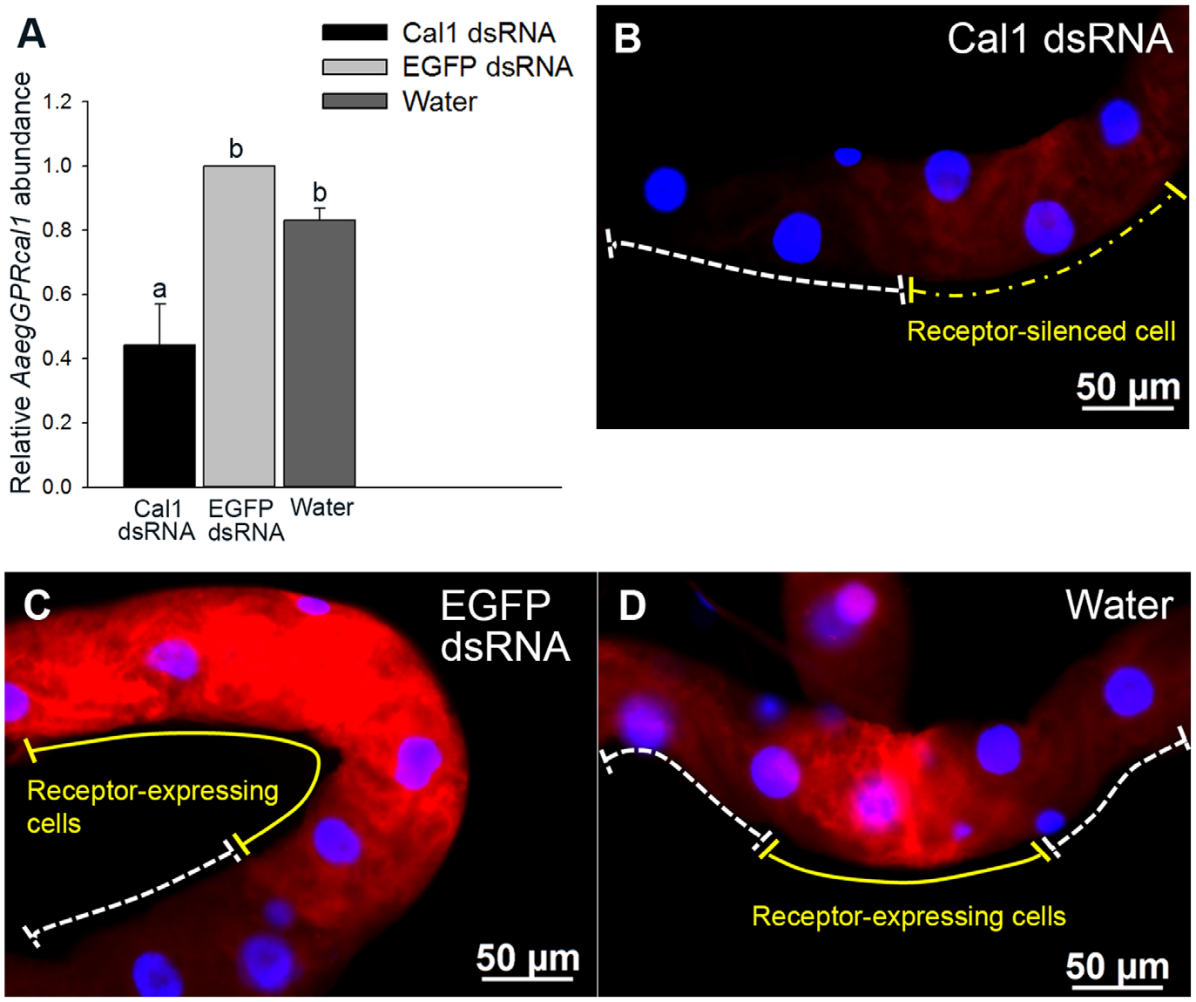
RNAi effect on *AaegGPRcal1* transcript and *Aaeg*GPRCAL1 expression in principal cells of female MTs. (**A**) Relative quantification of *AaegGPRcal1* transcript in the MTs five days after injection with *AaegGPRcal1* dsRNA, EGFP dsRNA or water. Bars represent mean ± S.D.; 1 indicates the calibrator. Data were analyzed by ANOVA followed by Tukey multiple comparison test (common letter indicates not significantly different at 0.05 level). (B–D) Fluorescence microscopy analyses; images were obtained with the same exposure time (200 msec). Females injected with *AaegGPRcal1* dsRNA exhibited lower *Aaeg*GPRCAL1 fluorescent signal intensity (**B**, *yellow dashed line*) by a factor of 3 than those injected with EGFP dsRNA (**C**, *yellow solid line*) or water (**D**, *yellow solid line*).

The receptor relative transcript abundance in females injected with *AaegGPRcal1* dsRNA was significantly reduced (∼47%) with respect to those injected with EGFP dsRNA or water as negative controls (Fig. 4A). In agreement, immunohistochemical analyses of female MTs from the RNAi experiments showed that *AaegGPRcal1* dsRNA treatment significantly reduced *Aaeg*GPRCAL1 signal intensity in the principal cells (Figs. 4B and S6). Pixel intensity of images from the RNAi treatment was reduced by a factor of ∼2 to 3 when compared to both controls (Figs. 4 B–D and S6).

**Figure 5:**
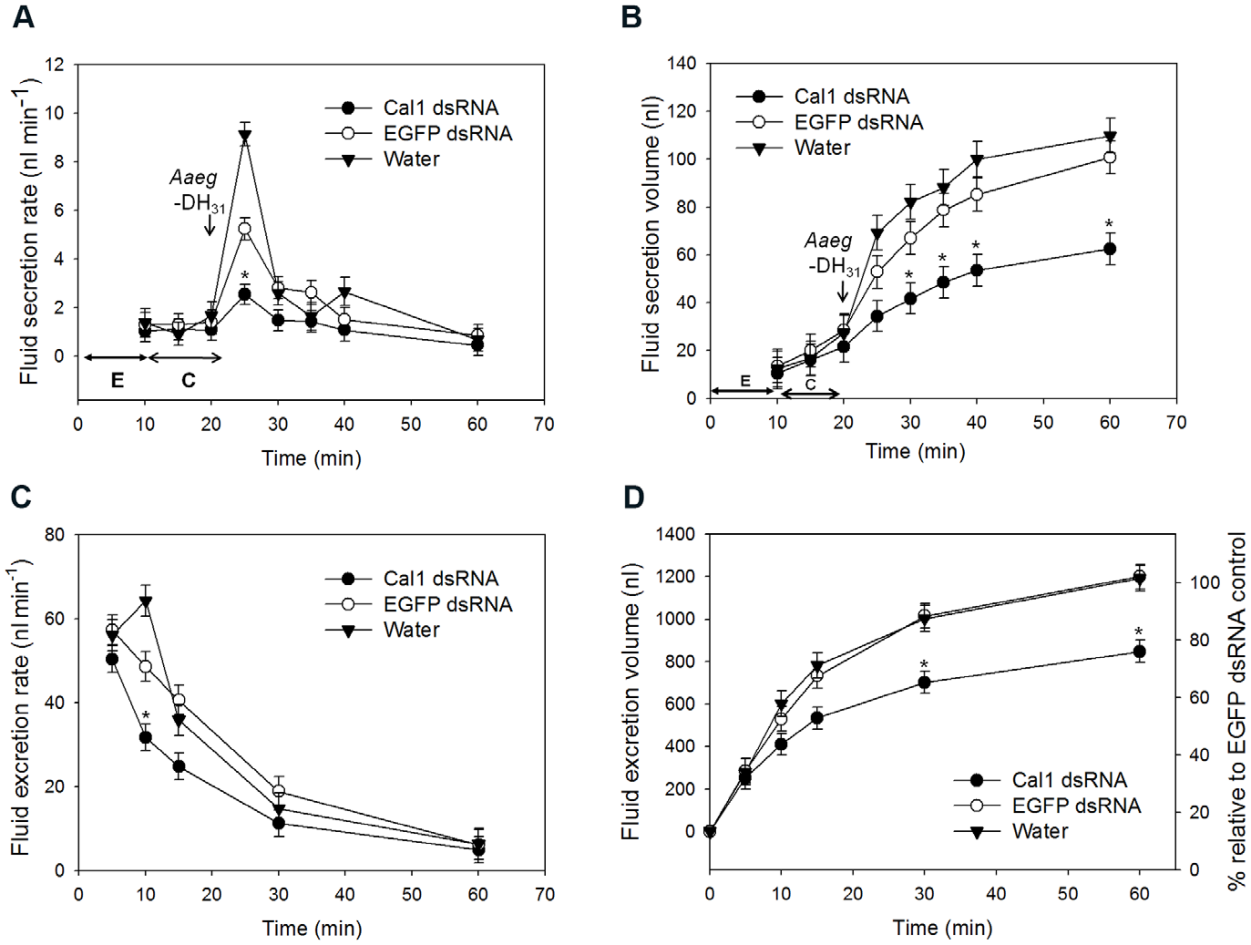
*AaegGPRcal1* knockdown effect on fluid secretion *in vitro* and excretion *in vivo*. (**A**) The rate of fluid secretion and secreted volume (**B**) were measured from single MTs from females treated with *AaegGPRcal1* dsRNA (n = 10), EGFP dsRNA (n = 9), or water (n = 9). In (**A)** time periods E (↔) and C (↔) indicate equilibration and control conditions, respectively. *In vivo* effect of RNAi on the rate of fluid excretion (**C**) and cumulative excreted volume (**D**) over 1 h from females injected with *AaegGPRcal1* dsRNA (*N* = 14), EGFP dsRNA (*N* = 11) or water (*N* = 10). Bars represent mean ± S.E.M (**A–D**) and data were analyzed with repeated measures using PROC GLMM Tukey-Kramer (* = *P<0.05*). In **A**, the * represent significant differences among all treatments only at 5 min after peptide application. In **B**–**D** the * represents only significant differences between the *AaegGPRcal1* dsRNA treatment and both controls; there are no significant differences between control treatments (**B**–**D**).

To evaluate the effect of receptor knock-down on the fluid secretion rate (nl/min), an *in vitro* fluid secretion assay modified after Ramsay [27] was performed in individual MTs from treated females (Fig. 5A, B). MTs from *AaegGPRcal1* dsRNA injected females exhibited a basal secretion rate similar to that of MTs from control females. A maximal rate of fluid secretion in MTs from all treatments was achieved 5 min after the addition of *Aaeg*-DH_31_ peptide to the medium (Fig. 5A, arrow). The DH_31_-stimulated maximal secretion rate achieved by MTs from the EGFP dsRNA and water treatments was higher by factors of 3.7 and 5.5, respectively, than during their respective control period C (Fig. 5A), while the stimulated rate in MTs from *AaegGPRcal1* dsRNA mosquitoes was only increased by a factor of 2.35 their basal secretion rate (Fig. 5A, asterisk; denotes significant differences among all treatments). For the *AaegGPRcal1* dsRNA injected females this represents a reduction in fluid secretion rate of 72% and 52% in comparison to those of the water and EGFP dsRNA treatments, respectively (Fig. 5A). After achieving the maximal secretion rate, rates decreased and remained low and similar for all groups (Fig. 5A). The higher maximum rate of fluid secretion from MTs treated with EGFP dsRNA and water resulted in higher final secreted volumes (Fig. 5B) than those of *AaegGPRcal1* dsRNA MTs; these significant differences between the *AaegGPRcal1* dsRNA and both controls were first detected 10 min after *Aaeg*-DH_31_ application and for the remaining 30 min (Fig. 5B, asterisks). There was no difference between controls. The total volume (100 nl) secreted per MT over 40 min after *Aaeg*-DH_31_ application in the controls (150 nl/h) (Fig. 5B) was similar to that found by other researchers (125 nl/h) [13].

To verify in live females the effect of *AaegGPRcal1* RNAi during post-prandial diuresis, treated females were confined in a precision humidity chamber after fully gorged with a blood meal. In agreement with the *in vitro* assays, the fluid excretion rate measured 5 to 10 min after blood feeding was significantly lower (*P*<0.05) in *AaegGPRcal1* dsRNA knocked down females than in those from both control treatments (Fig. 5C). The rates did not statistically differ between females in both control groups (Fig. 5C). The effect of the lower initial excretion rates in receptor-silenced females (Fig. 5C) is reflected in their lower cumulative fluid excretion volume that began to be significantly lower than both controls at 30 min (Fig. 5D, asterisks), with their total fluid loss over 1 h being reduced by 30% (Fig. 5D).

Together, these results indicate that the *AaegGPRcal1* dsRNA had a specific effect reducing *AaegGPRcal1* gene transcript and consequently, receptor protein, causing a significant decrease in the rate of fluid secretion in isolated renal organs in response to *Aaeg*-DH_31_, as well as from intact females immediately after a blood meal, resulting in a phenotype exhibiting reduced volume of fluid excreted.

## Discussion

In females of many species of mosquitoes blood feeding is necessary for egg development. It is also during blood feeding that pathogens are transmitted to humans and animals. *A. aegypti* is the vector of numerous arboviruses that cause morbidity and mortality. Females ingest in blood more than twice their unfed body weight, and nearly 40% of the water and salt ingested are excreted within 1–2 h after feeding [38]. Evolutionarily, this rapid fluid elimination may have developed to maintain ionic and water homeostasis and escaping predation by quickly eliminating added weight, regaining the ability to fly [39]. Altogether our results suggest that *Aaeg*GPRCAL1 is an important component of the successful adaptation to blood feeding in an anautogenous mosquito species, as it is responsible for the fast and increased fluid secretion post-blood meal [13].

Although the phylogenetic analysis does not resolve whether *Aaeg*GPRCAL1 is evolutionarily closer to hCALCRL or hCALCR, we concluded *Aaeg*GPRCAL1 is more similar to hCALCRL. This is also supported by: 1) the presence in *Aaeg*GPRCAL1 of functionally significant conserved residues that interact with hRAMPs in hCALCRL, 2) evidence that the orthologous fruit fly GPRCAL1 receptor requires co-expression of hRAMPs for activation [21] and, 3) that *Aaeg*GPRCAL1 is glycosylated, and receptor glycosylation is critical for hRAMPs interactions with hCALCRL [20]. Structural similarities between vertebrate CTs and *Aaeg*-DH_31_ (Fig. S4C) suggest some ligand structural features required for interaction with this class of receptors have been conserved.

*Aaeg*GPRCAL1 protein expression was confirmed by western blot analyses (Fig. 1A). The band at (73 kDa represents the glycosylated protein. We only observed one additional ∼51 kDa band, slightly above the 46.9 kDa predicted mass, that did not change in size upon treatment with PNGase F endoglycosidase. Thus, either the receptor runs as a 51 kDa band or the ∼4 kDa mass difference may result from either post-translational modifications at predicted phosphorylation sites or O-linked glycosylation at Ser/Thr residues (Fig. S1) [37], [40]. The presence of glycosylated and unglycosylated receptor forms in the MT suggests that the subpopulation of glycosylated receptors is structurally suitable for functional interaction with yet unknown proteins analogous to mammalian RAMPs, and that glycosylation may regulate receptor activation.

The MT hemolymph to lumen osmotic gradient is energized by the proton V-ATPase in the principal cells. The transepithelial secretion of Na^+^ and K^+^ depends on this proton electrochemical gradient because it is coupled to a proton/cation antiporter in the same membrane. Further, transport is also driven from the hemolymph through cotransporters, and ion channels. The resulting electrochemical gradient which is lumen positive then drives water and Cl^-^ passively from the hemolymph to the MT lumen generating primary urine [5], [41]. In response to *Aaeg*-DH_31_ an increase of cAMP activates Na^+^ channels and the Na^+^-K^+^-2Cl^−^ co-transporter in the basal membrane of principal cells [14] and up-regulates the V-ATPase function for increased fluid secretion [42]. Along the tubule, high V-ATPase expression is found in the distal principal cells [9]. Critical effectors such as the apical/subapical cation proton exchanger (*Ae*NHE8) and the basal sodium/proton exchanger (*Ae*NHE3) are also localized in the distal principal cells [43]–[45], with *Ae*NHE3 also present in proximal cells [45]. Among the diuretic hormones, *Aaeg*-DH_31_ is the only one that is natriuretic [1], and in agreement with the distal MT being the major secreting segment, principal cells in this region had the highest probability of expressing *Aaeg*GPRCAL1. The co-localization and high intensity of signal of *Aaeg*GPRCAL1, V-ATPase and exchangers in the secreting distal MTs may explain the rapid movement of ions, mainly sodium, and water in response to *Aaeg*-DH_31_. The novel, regional and variegated pattern of *Aaeg*GPRCAL1 spatial distribution of *Aaeg*GPRCAL1 found in principal cells along the MTs provides the mechanistic explanation for the differential regional secretory capacity of the tubule in response to *Aaeg*-DH_31_, revealing a more compartmentalized model for water and ion transport in this simple epithelium than previously thought [46].

Although the overall roles of principal and stellate cells in osmoregulation are understood, little is known about the transcriptional control of GPCRs regulating ion transport in specific principal cells. Unexpectedly, the receptor signal was detected in certain principal cells, but not all, even within the distal tubule (Figs. 1B, C, F, G and S5). Although the molecular and genetic mechanisms regulating *Aaeg*GPRCAL1 expression in certain principal cells are unknown, our results point to functionally different subtypes of phenotypically similar cells [47].

It is established that diuretic hormones induce fluid excretion within less than 2 min [14] post blood meal, and initial rates vary with hormone concentration which depends on blood meal size [48]. We showed this receptor regulates immediate fluid secretion from the MTs in response to *Aaeg*-DH_31_ application, and high excretion rates from intact females post blood meal (Fig. 5). Herein, both *in vitro* application of 1 µM *Aaeg*-DH_31_ to isolated MTs and a full blood meal given to females increased the rate of fluid secretion within 5 min, followed by a return to steady state (Fig. 5A, C). *In vitro*, only for the rate of fluid secretion of isolated MTs 5 min after hormone application, there were significant differences among all treatments (Fig. 5A). The rate of secretion in tubules from EGFP-injected mosquitoes could have been reduced with respect to those of water-injected mosquitoes due to the physiological cost of mounting an RNAi response. This may be similar to what has been demonstrated in the mammalian system, in which several genes were up- and down-regulated in response to the GFP construct [49]. Regardless, and most importantly, the isolated tubules from the EGFP-treatment had a statistically significantly higher rate of fluid secretion than those tubules in which *AaegGPRcal1* was silenced (Fig. 5A).

Other studies also found that MTs maximally stimulated for fluid secretion, either by *Anoga*-DH_31_ or cAMP had no further response to additional peptide or cAMP application [13], [50], similar to our findings. *In vitro*, the decrease of fluid secretion rate after 5 min of *Aaeg*-DH_31_ application (Fig. 5A) can be explained by the simultaneous activation of cAMP phosphodiesterase (PDE), a feedback mechanism to inhibit cAMP signaling [51]. Additionally, the involvement of G protein-coupled receptor kinases (GRKs) and β-arrestins for desensitization and internalization of the *Aaeg*GPRCAL1 receptor is likely [52], [53]. *In vivo*, the degradation of *Aaeg*-DH_31_ in hemolymph is thought to be extremely fast as shown for other blood sucking insects [14], explaining the decrease in fluid excretion rate for all treatments (Fig. 5C).

Previous studies in mosquitoes unequivocally showed that rapid fluid excretion during the peak phase is driven by the action of natriuretic hormones (MNF, *Anoga*-DH_31_) acting through cAMP to increase the sodium permeability of the basolateral and apical membranes of principal cells in a secretory direction [5], [13]. The peak phase maximal secretion rate of 56.5 nl/min measured 5 min post-blood meal (Fig. 5C) is similar to a previously reported of 54.4 nl/min [38]. The post-peak phase rate of 33.6 nl/min at 30 min post-blood meal was higher than the 11.1 nl/min previously reported for this phase [38]. The differences are likely due to our increased precision for measuring fluid excretion throughout all phases of diuresis.

Upon blood feeding, the high initial secretion rates reflect the high concentration of released *Aaeg*-DH_31,_ which stimulates *Aaeg*GPRCAL1 for maximal activation [48], [54]. We did not observe differences in fluid excretion rate among treatments in the first 5 min post blood meal (Fig. 5C). RNAi was not 100% effective in reducing receptor protein expression in the MTs [55]. The population of remaining receptors available in the *Aaeg*GPRCAL1 dsRNA treated females appears to suffice for the production of an initial normal response due to the initial high level of circulating hormone. The similarly decreasing rates in both control treatments (Fig. 5C) for the first 30 min suggest that this negative slope is controlled by the steady decrease in the hormone concentration in the hemolymph. In contrast, the rate of fluid excretion in the knock-down treatment is significantly reduced 10 min post-blood meal. Due to the lower expression level of receptors in the RNAi treatment, the fluid excretion rate is not sustained, therefore, a significant difference is clearly observed (Fig. 5C). This is perhaps compounded by a fast rate of receptor desensitization upon hormone application within the first 10 min, as shown clearly in the *in vitro* assay within 5 min (Fig. 5A). At the cellular level, the lower volume of primary urine results from lower receptor expression and the consequent decrease in signal transduction and amplification, reducing the downstream activation of effectors such as co-transporters, exchangers and channels. At the organismal level, as the fluid is excreted, the midgut distension decreases due to fluid transport from the midgut to the hemolymph, reducing the input for diuretic hormone release triggered by stretch receptors [14], [48]. Our RNAi results strongly support that *Aaeg*-DH_31_ activates *Aaeg*GPRCAL1 for immediate fluid excretion post blood meal (Fig. 5C, D).

In summary, this is the first comprehensive analysis of the physiological function of a family B GPCR in the regulation of diuresis in invertebrates. We demonstrated that *Aaeg*GPRCAL1 is not expressed in all principal cells in the renal organs, but rather in some perhaps specialized cells in the distal tubules, where the proton/cation exchangers in the basolateral membrane and the V-ATPase in the apical membrane are highly expressed. Therefore, the localization of *Aaeg*GPRCAL1 points to a compartmentalization of hormone signaling to achieve high rates of fluid transport, and the receptor unusual spatial expression is under control of a yet unknown mechanism. This simple epithelium offers a new model to further explore functional differences in phenotypically similar cells in renal organs.

## Supporting Information

Figure S1 ***AaegGPRcal1*** **full length cDNA cloned from MTs, and deduced amino acid sequence.** The cDNA sequence is 1995 bp, encoding a 412 amino acid residue protein. Seven transmembrane regions are predicted by TMHMM and underlined (•–•). The highly conserved six cysteine (C21, C40, C49, C63, C80, C102), two tryptophan (W50, W86), two proline (P51, P64), and aspartic acid (D45) residues in Family B GPCRs are indicated with white letters in black circles (residues at the N terminus). Three predicted N-linked glycosylation sites are double-underlined. Black squares indicate prediction of potential phosphorylation sites by protein-kinase A, D, and G.
(PDF)

Figure S2 **Amino acid sequence alignment of calcitonin receptor-like receptors.** *Aaeg*GPRCAL1 was aligned with those of other arthropods, a mollusk, and vertebrates. The *A*. *aegypti* GPRCAL1(AEU12191)**^1^** sequence is 79% identical to *C. quinquefasciatus* GPRCAL1 (CPIJ014419–PA)**^2^**, 75% identical to *A. gambiae* GPRCAL1 (AGAP009770-PA)**^3^**, 64% to sequenced *D. melanogaster* GPRCAL1 (AAN16138)**^4^**, 55% to *P. humanus corporis* GPRCAL1 (PHUM428070-PA)**^5^**, 59% to *Nasonia vitripennis* GPRCAL1 (XP_001601649)**^6^**, 33% to human CALCLR (NP_005786)**^7^**, 33% to rat *R. norvegicus* CALCRL (NP_036849)**^8^**, 32% to chicken *G. gallus* CALCRL (NP_001157122)**^9^**, 32% to frog *X. laevis* CALCRL (NP_001080206)**^10^**, 34% to *D. rerio* CALCRLA (NP_001004010)**^11^**, 31% to *P. olivaceus* CGRPR (BAA92817)**^12^** and 27% to *C. gigas* CTR (CAD82836)**^13^**. Accession numbers in parenthesis are of putative (2–3 and 5–6) or cloned translated sequences (1, 4 and 7–13) from GenBank or VectorBase. Predicted transmembrane domains (TM) of *Aaeg*GPRCAL1 are indicated by a line above the sequences. Blastp analysis of the *Anopheles* genome with the *Aedes* receptor sequence identified the prediction of the *AgamGPRcal1* ORF, permitting the localization of intron-exon boundaries in the genome by eye gazing because the gene organization is also conserved in *Anopheles*. **Conserved residues between** ***Aaeg*** **GPRCAL1 and hCALCRL:** Residues in *Aaeg*GPRCAL1 with demonstrated functional significance in hCALCRL are as follows: receptor coupling with Gs (R146), receptor cell-surface expression (Y209, L210, H211, E371, and V372), structural stabilization (P236, P273, P323 and P333), GPCR kinases phosphorylation (S391, S398, T382, T387, T389 and T395). In hCALCRL, aspartate (D69) in the N-terminus and leucine (L351) in TM6 are important residues associated with RAMP1; these corresponding residues are conserved in *Aaeg*GPRCAL1 (D45 and L331) and *Dmel*GPRCAL1 (D73 and L360).
(PDF)

Figure S3 **Phylogenetic tree** **showing evolutionary relationships for GPRCAL1.** Corticotropin-releasing hormone 1-like receptors (CRHR1), CALCRs and CALCRLs from vertebrates and invertebrates were analyzed by the neighbor-joining method with bootstrap analysis of 10,000 replicates with MEGA5. A secretin type GPCR from *Caenorhabditis elegans* (NP_510496.1; WormBase WP:CE23557) was used as the outgroup to root tree. GenBank and VectorBase accession numbers: 1. *A. aegypti* GPRDIH1 (ABX57919). 2. *A. gambiae* GPRDIH1 (AGAP005464-PA). 3. *C. quinquefasciatus* GPRDIH1 (DAA06284). 4. *D. melanogaster* GPRDIH1 (AAF58250). 5. human CRHR1 (NP_001138618). 6. *Rattus norvegicus* CRHR1 (NP_112261). 7. *Gallus gallus* CRHR1 (AAA96656). 8. *Xenopus laevis* CRHR1 (CAA74363). 9. *A. aegypti* GPRCAL1 (AEU12191, this work). 10. *C. quinquefasciatus* GPRCAL1 (CPIJ014419–RA). 11. *A. gambiae* GPRCAL1 (AGAP009770-RA). 12. *D. melanogaster* GPRCAL1 (AAN16138). 13. *Pediculus humanus corporis* GPRCAL1 (PHUM428070). 14. *Nasonia vitripennis* GPRCAL1 (XP_001601649). 15. hCALCRL (NP_005786). 16. *R*. *norvegicus* CALCRL (NP_036849). 17. *G*. *gallus* CALCRL (NP_001157122). 18. *X*. *laevis* CALCRL (NP_001080206). 19. *Danio rerio* CALCRLA (NP_001004010). 20. *Paralichthys olivaceus* CGRPR (BAA92817). 21. hCALCR (AAC50300). 22. *R*. *norvegicus* CALCR (AAA03030). 23. *G*. *gallus* CALCR (XP_425985.3). 24. *D*. *rerio* (XP_003200679). 25. *Grassostrea gigas* CTR (CAD82836). Predicted proteins: 2–3, 10–11, 13–14 and 23–24. Protein sequences translated from cloned cDNAs: 1, 4–9, 12,15–22 and 25.
(PDF)

Figure S4 **Amino acid sequence alignment of calcitonins (CTs) and calcitonin gene related peptides (CGRPs).** (**A**) The *A. aegypti* diuretic hormone 31 (*Aaeg-*DH_31_) sequence was aligned with those of other arthropod DH_31_ and CTs and in (**B**) with CGRPs. In (**A**) and (**B**), GenBank accession numbers: 1. *A*. *aegypti* DH_31_ (EAT40182). 2. *A. gambiae* DH_31_ (XP_321755). 3. *D. melanogaster* (AAF52685). 4. *R. prolixus* DH_31_ (ACX47068). 5. *Bombyx mori* DH_31_ (NP_001124379). 6. *Apis mellifera* DH_31_ (P85830). 7. *N. vitripennis* DH_31_ (XP_001599948). 8. *T. castaneum* DH_31_ (EEZ99367). 9. *D. punctata* DH_31_ (P82372). 10. *Acyrthosiphon pisum* DH_31_ (XP_001945901). 11. hCT (AAA58403). 12. hCGRP (1005250A). 13. *R. norvegicus* CT (AAA40849). 14. *R. norvegicus* CGRP (NP_612522). 15. *G. gallus* CT (ABY65359) 16. *G. gallus* CGRP (P10286). 17. *Samo salar* CT (NP_001135058). 18. *S*. *salar* CGRP (NP_001140052). Predicted hormone sequences: 2–3, 6–8 and 10; translated from cloned cDNAs: 1, 4–5, 9, 11–18. (**C**) Predicted protein structure of the *Aaeg*-DH_31_. Amino acid residues in pink are conserved in human CT.
(PDF)

Figure S5 **Immunolocalization of** ***Aaeg*** **GPRCAL1 along the length of a single MT.** The receptor signal (red, white arrows) was observed in only particular principal cells, the majority located towards the distal end which contains the tip cell.
(PDF)

Figure S6 **Localization of** ***Aaeg*** **GPRCAL1 in MTs from females injected with** ***AaegGPRcal1*** **dsRNA, EGFP dsRNA and water.** (**A**) MTs from *AaegGPRcal1* knock-down females exhibited reduction of receptor signal intensity (in this image by a factor of 2) compared to those of controls (**B**) EGFP dsRNA and (**C**) water.
(PDF)

Table S1 **Primers used for cloning, transcriptional and functional analyses (RNAi) of** ***AaegGPRcal1.***
(PDF)
