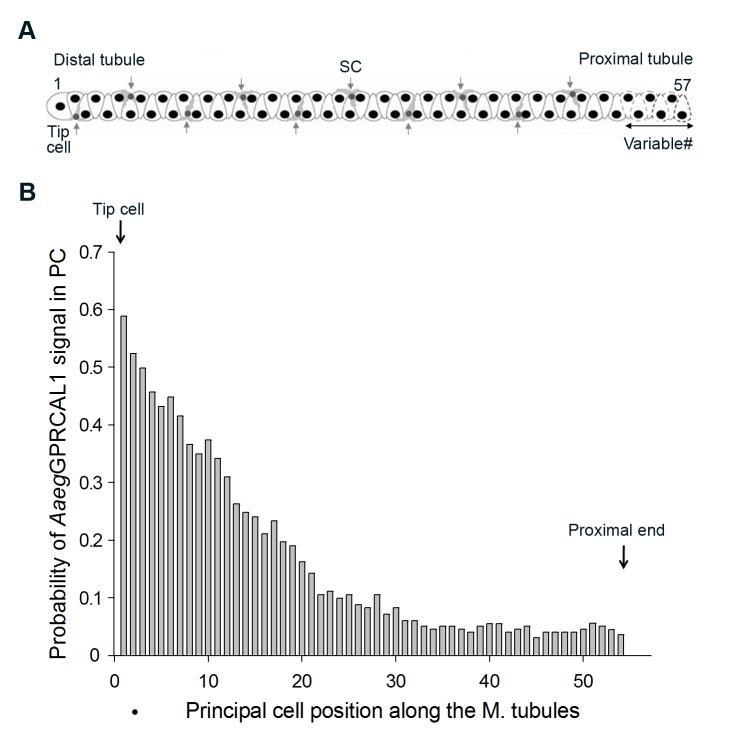# Correction: Role in Diuresis of a Calcitonin Receptor (GPRCAL1) Expressed in a Distal-Proximal Gradient in Renal Organs of the Mosquito *Aedes aegypti* (L.)

**DOI:** 10.1371/annotation/9d100bb6-b5ee-4fd0-81e9-c315523e7f75

**Published:** 2013-10-23

**Authors:** Hyeogsun Kwon, Hsiao-Ling Lu, Michael T. Longnecker, Patricia V. Pietrantonio

The Y-axis of Figure 3 was incorrectly scaled. You may see the corrected Figure 3 here: 

**Figure:**