# Supplementary material for: Role in Diuresis of a Calcitonin Receptor (GPRCAL1) Expressed in a Distal-Proximal Gradient in Renal Organs of the Mosquito Aedes aegypti (L.)
Source: PLoS One. 2012 Nov 29;7(11):e50374. doi: 10.1371/journal.pone.0050374 (PMC3510207; doi:10.1371/journal.pone.0050374)
Supplement: Figure S1 — AaegGPRcal1 full length cDNA cloned from MTs, and deduced amino acid sequence. The cDNA sequence is 1995 bp, encoding a 412 amino acid residue protein. Seven transmembrane regions are predicted by TMHMM and underlined (•–•). The highly conserved six cysteine (C21, C40, C49, C63, C80, C102), two tryptophan (W50, W86), two proline (P51, P64), and aspartic acid (D45) residues in Family B GPCRs are indicated with white letters in black circles (residues at the N terminus). Three predicted N-linked glycosylation sites are double-underlined. Black squares indicate prediction of potential phosphorylation sites by protein-kinase A, D, and G. (PDF) [file pone.0050374.s001.pdf]

ATTCTTTGCCAGATATCAACCAAACACACAAACCCAGTTTAGCCACCAGTAGTAGTAG -37

GAGGAACGATACGTCCTCAAACCAAAATAAAAAAAA**ATG**ACATCCTCAACAACGACGGACGCA 27

M T S S T T T D A 9

ACCGATTCGGAGTTCGACCAGAGGAAGCTTCAATGCTTGGAAATGCTCAACGCAACCACCGAA 90

T D S E F D Q R K L Q C L E M L N A T T E 30

TTCACCACAACCCGGTCTGGACCTTTCTGCAGGGGAACCTGGGATGGTTGGCTCTGTTGGCCG 153

F T T T R S G P F C R G T W D G W L C W P 51

GATACTGCGGCTGGCGAATCTGCACTGCTACCCTGCCCAGATTTCATGGATGGATTCGATCCA 216

D T A A G E S A L L P C P D F M D G F D P 72

ACAAGATTCGCACACAAAGACTGCGATGAGGACGGAGAATGGTTTAGGCATCCACTGACCAAC 279

T R F A H K D C D E D G E W F R H P L T N 93

AGAACTTGGTCCAATTACACAACCTGCGTTAATTTGGATAAGCTCGAGTGGATGGAACAAGTG 342

R T W S N Y T T C V N L D K L E W M E Q V 114

AGAACGATATACGAAACGGGATACTCGATTTCGCTGATAGCCCTCATCTTATCGCTTGGTATT 405

R T I Y E T G Y S I S L I A L I L S L G I 135

TTAAGTTACTTTAGGTCACTGAAATGCGCTCGCATTACGCTACATATGAACCTGTTTGCGTCG 468

L S Y F R S L K C A R I T L H M N L F A S 156

TTTGCCTCAAACAACACACTCTGGCTGCTGTGGTACCGGATGGTGCTGGCAGATCCGGAAGTT 531

F A S N N T L W L L W Y R M V L A D P E V 177

CTCAAGCACAATGGGGCGTCCTGCATCACACTGCACCTGGTGCTGCACTACTTCCTCATCACC 594

L K H N G A S C I T L H L V L H Y F L I T 198

AACTACGCGTGGATGCTCTGCGAAGGATTCTATCTGCACACGGTCCTGGTGTCGGCATTCGTT 657

N Y A W M L C E G F Y L H T V L V S A F V 219

TCGGAGAAAAAACTCGTCAACTGGCTTGTGGTCCTCGGATGGACCACACCGGGGATCGTGATC 720

S E K K L V N W L V V L G W T T P G I V I 240

ATGGCGTATGGATTTCTGCGAGGATACGCCGGTACCCCGGAGGATACGATAGAGTGCTGGATG 783

M A Y G F L R G Y A G T P E D T I E C W M 261

AACGAAAGCGTGTATGATAACGTTTTCAAGGCACCGGTGTGCATCTCGATGCTGCTCAATTTG 846

N E S V Y D N V F K A P V C I S M L L N L 282

CTATTCCTGTGCAATATCATACGAGTAGTTCTGCTGAAGTTGAAAGCTCCTGCGGGACCGCAA 909

L F L C N I I R V V L L K L K A P A G P Q 303

GGTACCGGGCCATCGAGAACCATACTGCAAGCGTTCAGAGCCACTCTGCTGCTAGTTCCGCTT 972

G T G P S R T I L Q A F R A T L L L V P L 324

CTTGGCCTACAGTACATATTGACTCCGTTCCGACCCGATCCTGGACATTCTTATGAGCGAACG 1035

L G L Q Y I L T P F R P D P G H S Y E R T 345

TACGAAATTATATCGGCGTTCACGGCATCATTCCAAGGTCTATTCGTAGCCGTCCTGTTCTGT 1098

Y E I I S A F T A S F Q G L F V A V L F C 366

TTCTTCAACGGAGAGGTTATCGCACAAGTGAAACGGAAATGGCGCACCGTGTTCTTGCGGACT 1161

F F N G E V I A Q V K R K W R T V F L R T 387

```
CGAACCAATTCCTACACCGCAACGCAAGTGTCCTTCATTCGTAGCGGTCCGCCTATTCCCGGC     1224
 R  T  N  S  Y  T  A  T  Q  V  S  F  I  R  S  G  P  P  I  P  G      408

GAAGAGAAGGTCTGACTAAAAGAGTACGCCAGCGGCAATAGGTCTCCCCAGCCAACGAGCTTG     1287
 E  E  K  V  *                                                      412
AACCCCGTTAACGATCGGAGTTTTTCGGGAAATCCAACGACACCAACGGGTTTGGAAGGTAAG     1350
AGCAACGGTAATGGACCGGACTTATCGGTAGACGTGTCGAAAGATGACGCAATCTTTGGAGAC     1413
GCCAAAGCAAAGATCAATGGAGCTTCCGCCCATATCGAAGAACGGCTCGGCACATGGCGGAGA     1476
GGTTCAATTGAGTGTAGACATGTGAAGTTACCTCTTCTGGAAGCGCAACACAGGGAAAACGAT     1539
GGGGATCGTGAGGGAGAGGCTGAACAAGCTTCGCAACTTTTCGTTGAGCCGGCTCCGGCAGAT     1602
GAAGTGTTGTGAACTCTCGTTAGAACACAAATGTATCGAATTAATGATAGGTGAATTTATTAG     1665
CTTAAGGTTCAAAGAATTTCGAATATGTACAATGATGTAGTCATTCTAAGCTTAGTCTTACTA     1728
TTGTGACCGGTGGAAACCCACTTAACTACGCGTAGTTTTTATTTTAACGTTCTTAAACGCTTC     1791
AATTTTTTTGTAGCAAATACACAAACACACTCCACCTGTTAAGGGATAAATGTGATCATGGTG     1854
CAAAATAAAACTATCTGATTCGTGAAAAAAAAAAAAAAAAAAAAAA                      1901
```

Figure S1. *AaegGPRcal1* full length cDNA cloned from MTs, and deduced amino acid sequence. The cDNA sequence is 1995 bp, encoding a 412 amino acid residue protein. Seven transmembrane regions are predicted by TMHMM and underlined (●——●). The highly conserved six cysteine (C21, C40, C49, C63, C80, C102), two tryptophan (W50, W86), two proline (P51, P64), and aspartic acid (D45) residues in Family B GPCRs are indicated with white letters in black circles (residues at the N terminus) [1,2]. Three predicted N-linked glycosylation sites are double-underlined. Black squares indicate prediction of potential phosphorylation sites by protein-kinase A, D, and G.

References

1. Harmar AJ (2001) Family-B G-protein-coupled receptors. Genome Biol 2: 3013.

2. Hoare SR (2005) Mechanisms of peptide and nonpeptide ligand binding to Class B G-protein coupled receptors. Drug Discov Today 10: 417-427.
