# Supplementary material for: Role in Diuresis of a Calcitonin Receptor (GPRCAL1) Expressed in a Distal-Proximal Gradient in Renal Organs of the Mosquito Aedes aegypti (L.)
Source: PLoS One. 2012 Nov 29;7(11):e50374. doi: 10.1371/journal.pone.0050374 (PMC3510207; doi:10.1371/journal.pone.0050374)
Supplement: Figure S2 — Amino acid sequence alignment of calcitonin receptor-like receptors. AaegGPRCAL1 was aligned with those of other arthropods, a mollusk, and vertebrates. The A. aegypti GPRCAL1(AEU12191)1 sequence is 79% identical to C. quinquefasciatus GPRCAL1 (CPIJ014419–PA)2, 75% identical to A. gambiae GPRCAL1 (AGAP009770-PA)3, 64% to sequenced D. melanogaster GPRCAL1 (AAN16138)4, 55% to P. humanus corporis GPRCAL1 (PHUM428070-PA)5, 59% to Nasonia vitripennis GPRCAL1 (XP_001601649)6, 33% to human CALCLR (NP_005786)7, 33% to rat R. norvegicus CALCRL (NP_036849)8, 32% to chicken G. gallus CALCRL (NP_001157122)9, 32% to frog X. laevis CALCRL (NP_001080206)10, 34% to D. rerio CALCRLA (NP_001004010)11, 31% to P. olivaceus CGRPR (BAA92817)12 and 27% to C. gigas CTR (CAD82836)13. Accession numbers in parenthesis are of putative (2–3 and 5–6) or cloned translated sequences (1, 4 and 7–13) from GenBank or VectorBase. Predicted transmembrane domains (TM) of AaegGPRCAL1 are indicated by a line above the sequences. Blastp analysis of the Anopheles genome with the Aedes receptor sequence identified the prediction of the AgamGPRcal1 ORF, permitting the localization of intron-exon boundaries in the genome by eye gazing because the gene organization is also conserved in Anopheles. Conserved residues between Aaeg GPRCAL1 and hCALCRL: Residues in AaegGPRCAL1 with demonstrated functional significance in hCALCRL are as follows: receptor coupling with Gs (R146), receptor cell-surface expression (Y209, L210, H211, E371, and V372), structural stabilization (P236, P273, P323 and P333), GPCR kinases phosphorylation (S391, S398, T382, T387, T389 and T395). In hCALCRL, aspartate (D69) in the N-terminus and leucine (L351) in TM6 are important residues associated with RAMP1; these corresponding residues are conserved in AaegGPRCAL1 (D45 and L331) and DmelGPRCAL1 (D73 and L360). (PDF) [file pone.0050374.s002.pdf]

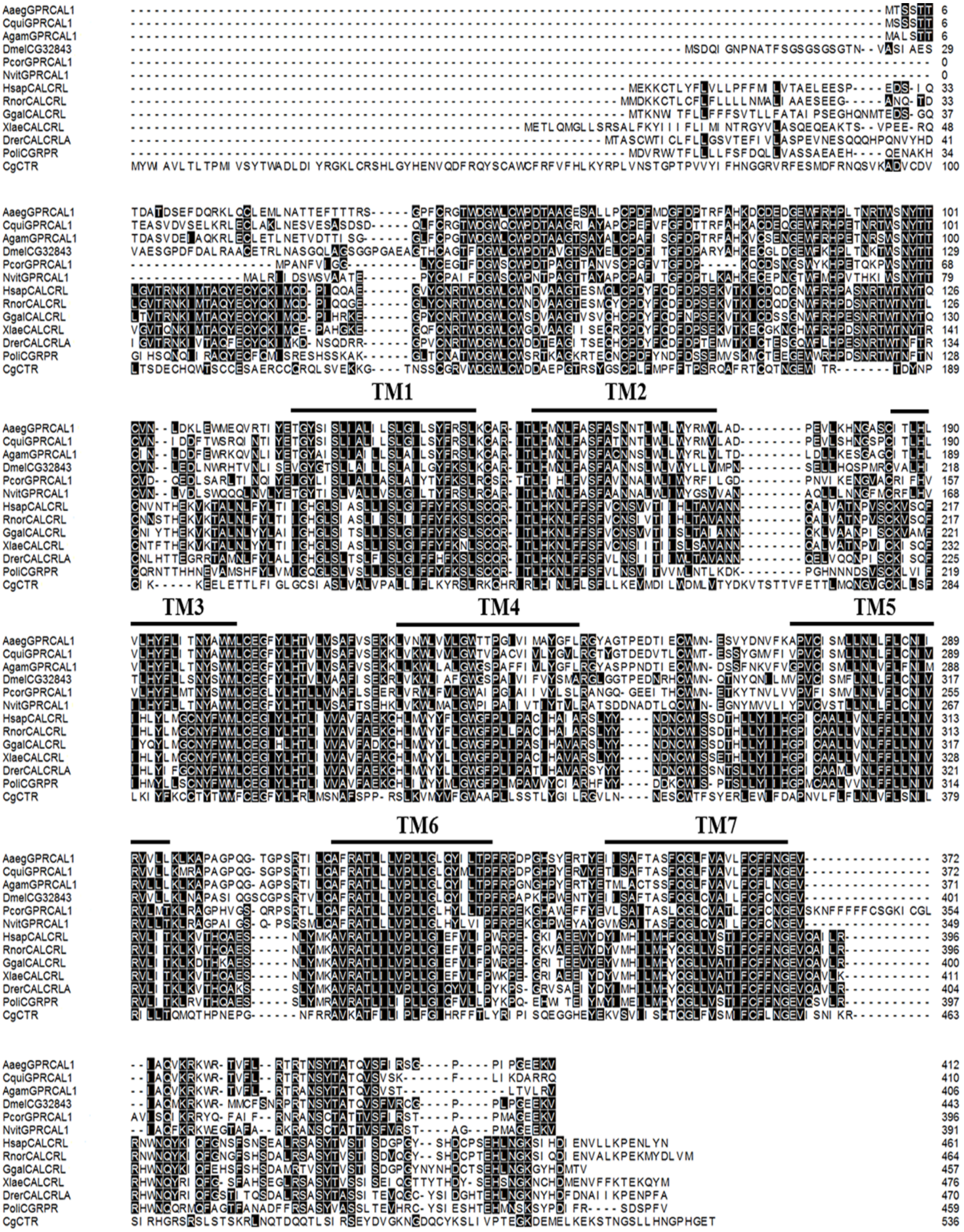
AaegGPRCAL1
CquiGPRCAL1
AgamGPRCAL1
DmelCG32843
PcorGPRCAL1
NvitGPRCAL1
HsapCALCRL
RnorCALCRL
GgalCALCRL
XlaeCALCRL
DrerCALCRLA
PoliCGRPR
CgCTR
TM1
TM2
TM3
TM4
TM5
TM6
TM7

Figure S2. Amino acid sequence alignment of calcitonin receptor-like receptors. *Aaeg*GPRCAL1 was aligned with those of other arthropods, a mollusk, and vertebrates. The *A. aegypti* GPRCAL1(AEU12191)[1] sequence is 79% identical to *C. quinquefasciatus* GPRCAL1 (CPIJ014419–PA)[2], 75% identical to *A. gambiae* GPRCAL1 (AGAP009770-PA)[3], 64% to sequenced *D. melanogaster* GPRCAL1 (AAN16138)[4] , 55% to *P. humanus corporis* GPRCAL1 (PHUM428070-PA)[5], 59% to *Nasonia vitripennis* GPRCAL1 (XP_001601649)[6], 33% to human CALCLR (NP_005786)[7], 33% to rat *R. norvegicus* CALCRL (NP_036849)[8], 32% to chicken *G. gallus* CALCRL (NP_001157122)[9], 32% to frog *X. laevis* CALCRL (NP_001080206)[10], 34% to *D. rerio* CALCRLA (NP_001004010)[11], 31% to *P. olivaceus* CGRPR (BAA92817)[12] and 27% to *C. gigas* CTR (CAD82836)[13]. Accession numbers in parenthesis are of putative (2-3 and 5-6) or cloned translated sequences (1, 4 and 7-13) from GenBank or VectorBase. Predicted transmembrane domains (TM) of *Aaeg*GPRCAL1 are indicated by a line above the sequences. Blastp analysis of the *Anopheles* genome with the *Aedes* receptor sequence identified the prediction of the *AgamGPRcal1* ORF, permitting the localization of intron-exon boundaries in the genome by eye gazing because the gene organization is also conserved in *Anopheles*. **Conserved residues between *Aaeg*GPRCAL1 and hCALCRL:** Residues in *Aaeg*GPRCAL1 with demonstrated functional significance in hCALCRL are as follows: receptor coupling with Gs (R146), receptor cell-surface expression (Y209, L210, H211, E371, and V372) [1,2], structural stabilization (P236, P273, P323 and P333) [3], GPCR kinases phosphorylation (S391, S398, T382, T387, T389 and T395) [4]. In hCALCRL, aspartate (D69) in the N-terminus and leucine (L351) in TM6 are important residues associated with RAMP1; these corresponding residues are conserved in *Aaeg*GPRCAL1 (D45 and L331) and *Dmel*GPRCAL1 (D73 and L360) [5,6].

References

1. Conner AC, Simms J, Conner MT, Wootten DL, Wheatley M, et al. (2006) Diverse functional motifs within the three intracellular loops of the CGRP1 receptor. Biochemistry 45: 12976-12985.

2. Conner M, Hicks MR, Dafforn T, Knowles TJ, Ludwig C, et al. (2008) Functional and biophysical analysis of the C-terminus of the CGRP-receptor; a family B GPCR. Biochemistry 47: 8434-8444.

3. Conner AC, Hay DL, Simms J, Howitt SG, Schindler M, et al. (2005) A key role for transmembrane prolines in calcitonin receptor-like receptor agonist binding and signalling: implications for family B G-protein-coupled receptors. Mol Pharmacol 67: 20-31.

4. Kuwasako K, Kitamura K, Nagata S, Hikosaka T, Kato J (2010) Function of the cytoplasmic tail of human calcitonin receptor-like receptor in complex with receptor activity-modifying protein 2. Biochem Biophys Res Commun 392: 380-385.

5. Barwell J, Conner A, Poyner DR (2011) Extracellular loops 1 and 3 and their associated transmembrane regions of the calcitonin receptor-like receptor are needed for CGRP receptor function. Biochim Biophys Acta 1813: 1906-1916.

6. Ittner LM, Luessi F, Koller D, Born W, Fischer JA, et al. (2004) Aspartate69 of the calcitonin-like receptor is required for its functional expression together with receptor-activity-modifying proteins 1 and -2. Biochem Biophys Res Commun 319: 1203-1209.
