# Supplementary material for: Role in Diuresis of a Calcitonin Receptor (GPRCAL1) Expressed in a Distal-Proximal Gradient in Renal Organs of the Mosquito Aedes aegypti (L.)
Source: PLoS One. 2012 Nov 29;7(11):e50374. doi: 10.1371/journal.pone.0050374 (PMC3510207; doi:10.1371/journal.pone.0050374)
Supplement: Figure S3 — Phylogenetic tree showing evolutionary relationships for GPRCAL1. Corticotropin-releasing hormone 1-like receptors (CRHR1), CALCRs and CALCRLs from vertebrates and invertebrates were analyzed by the neighbor-joining method with bootstrap analysis of 10,000 replicates with MEGA5. A secretin type GPCR from Caenorhabditis elegans (NP_510496.1; WormBase WP:CE23557) was used as the outgroup to root tree. GenBank and VectorBase accession numbers: 1. A. aegypti GPRDIH1 (ABX57919). 2. A. gambiae GPRDIH1 (AGAP005464-PA). 3. C. quinquefasciatus GPRDIH1 (DAA06284). 4. D. melanogaster GPRDIH1 (AAF58250). 5. human CRHR1 (NP_001138618). 6. Rattus norvegicus CRHR1 (NP_112261). 7. Gallus gallus CRHR1 (AAA96656). 8. Xenopus laevis CRHR1 (CAA74363). 9. A. aegypti GPRCAL1 (AEU12191, this work). 10. C. quinquefasciatus GPRCAL1 (CPIJ014419–RA). 11. A. gambiae GPRCAL1 (AGAP009770-RA). 12. D. melanogaster GPRCAL1 (AAN16138). 13. Pediculus humanus corporis GPRCAL1 (PHUM428070). 14. Nasonia vitripennis GPRCAL1 (XP_001601649). 15. hCALCRL (NP_005786). 16. R. norvegicus CALCRL (NP_036849). 17. G. gallus CALCRL (NP_001157122). 18. X. laevis CALCRL (NP_001080206). 19. Danio rerio CALCRLA (NP_001004010). 20. Paralichthys olivaceus CGRPR (BAA92817). 21. hCALCR (AAC50300). 22. R. norvegicus CALCR (AAA03030). 23. G. gallus CALCR (XP_425985.3). 24. D. rerio (XP_003200679). 25. Grassostrea gigas CTR (CAD82836). Predicted proteins: 2–3, 10–11, 13–14 and 23–24. Protein sequences translated from cloned cDNAs: 1, 4–9, 12,15–22 and 25. (PDF) [file pone.0050374.s003.pdf]

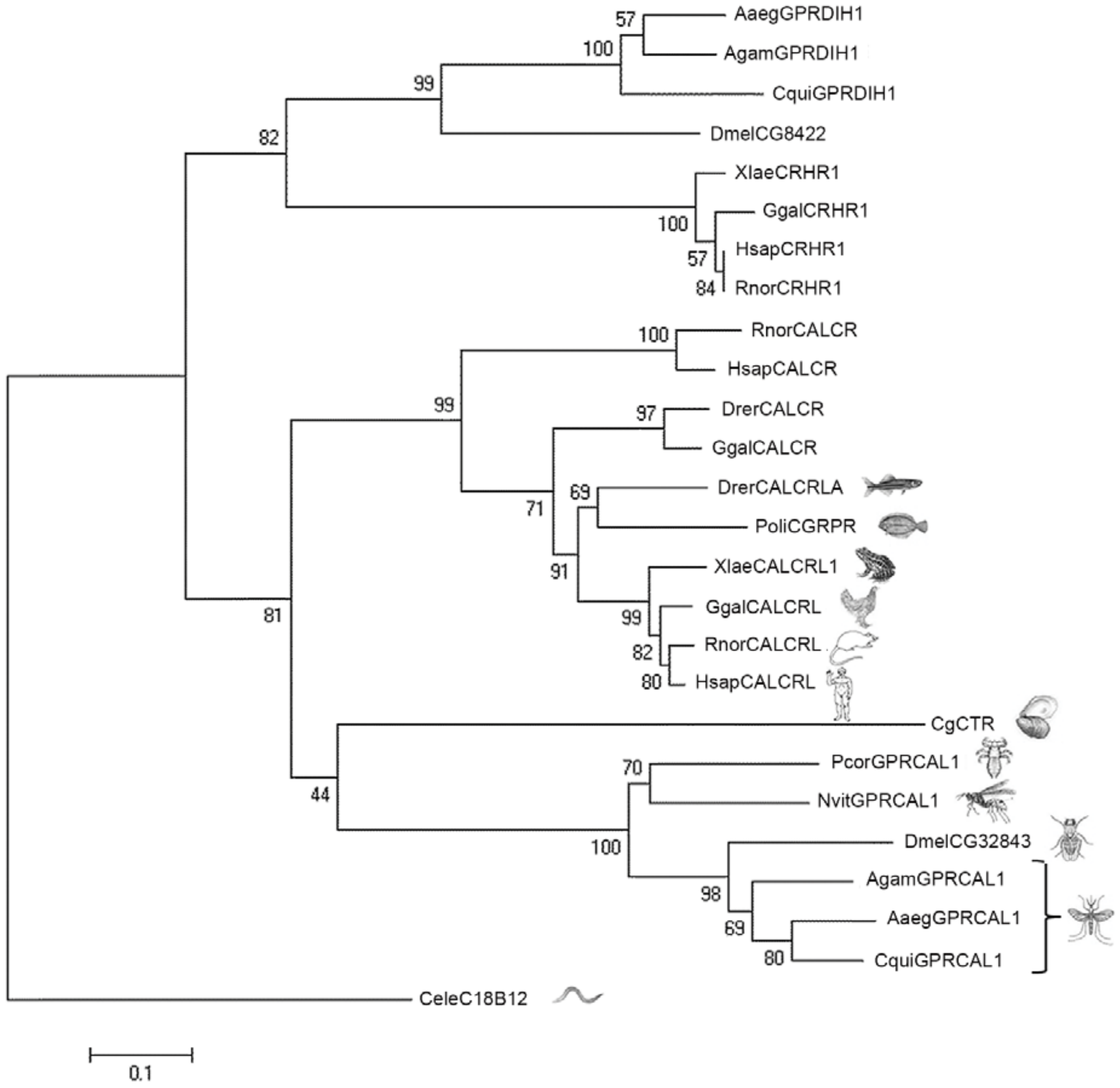

AaegGPRDIH1
AgamGPRDIH1
CquiGPRDIH1
DmelCG8422
XlaeCRHR1
GgalCRHR1
HsapCRHR1
RnorCRHR1
RnorCALCR
HsapCALCR
DrerCALCR
GgalCALCR
DrerCALCRLA
PoliCGRPR
XlaeCALCRL1
GgalCALCRL
RnorCALCRL
HsapCALCRL
CgCTR
PcorGPRCAL1
NvitGPRCAL1
DmelCG32843
AgamGPRCAL1
AaegGPRCAL1
CquiGPRCAL1
CeleC18B12
57
100
99
82
100
57
84
100
99
97
69
71
91
99
82
80
81
44
70
100
98
69
80
0.1

Figure S3. Phylogenetic tree showing evolutionary relationships for GPRCAL1. Corticotropin-releasing hormone 1-like receptors (CRHR1), CALCRs and CALCRLs from vertebrates and invertebrates were analyzed by the neighbor-joining method with bootstrap analysis of 10,000 replicates with MEGA5. A secretin type GPCR from *Caenorhabditis elegans* (NP_510496.1; WormBase WP:CE23557) was used as the outgroup to root tree. GenBank and VectorBase accession numbers: 1. *A. aegypti* GPRDIH1 (ABX57919). 2. *A. gambiae* GPRDIH1 (AGAP005464-PA). 3. *C. quinquefasciatus* GPRDIH1 (DAA06284). 4. *D. melanogaster* GPRDIH1 (AAF58250). 5. human CRHR1 (NP_001138618). 6. *Rattus norvegicus* CRHR1 (NP_112261). 7. *Gallus gallus* CRHR1 (AAA96656). 8. *Xenopus laevis* CRHR1 (CAA74363). 9. *A. aegypti* GPRCAL1 (AEU12191, this work). 10. *C. quinquefasciatus* GPRCAL1 (CPIJ014419–RA). 11. *A. gambiae* GPRCAL1 (AGAP009770-RA). 12. *D. melanogaster* GPRCAL1 (AAN16138). 13. *Pediculus humanus corporis* GPRCAL1 (PHUM428070). 14. *Nasonia vitripennis* GPRCAL1 (XP_001601649). 15. hCALCRL (NP_005786). 16. *R. norvegicus* CALCRL (NP_036849). 17. *G. gallus* CALCRL (NP_001157122). 18. *X. laevis* CALCRL (NP_001080206). 19. *Danio rerio* CALCRLA (NP_001004010). 20. *Paralichthys olivaceus* CGRPR (BAA92817). 21. hCALCR (AAC50300). 22. *R. norvegicus* CALCR (AAA03030). 23. *G. gallus* CALCR (XP_425985.3). 24. *D. rerio* (XP_003200679). 25. *Grassostrea gigas* CTR (CAD82836). Predicted proteins: 2-3, 10-11, 13-14 and 23-24. Protein sequences translated from cloned cDNAs: 1, 4-9, 12,15-22 and 25.
