# Supplementary material for: Role in Diuresis of a Calcitonin Receptor (GPRCAL1) Expressed in a Distal-Proximal Gradient in Renal Organs of the Mosquito Aedes aegypti (L.)
Source: PLoS One. 2012 Nov 29;7(11):e50374. doi: 10.1371/journal.pone.0050374 (PMC3510207; doi:10.1371/journal.pone.0050374)
Supplement: Figure S4 — Amino acid sequence alignment of calcitonins (CTs) and calcitonin gene related peptides (CGRPs). (A) The A. aegypti diuretic hormone 31 (Aaeg-DH31) sequence was aligned with those of other arthropod DH31 and CTs and in (B) with CGRPs. In (A) and (B), GenBank accession numbers: 1. A. aegypti DH31 (EAT40182). 2. A. gambiae DH31 (XP_321755). 3. D. melanogaster (AAF52685). 4. R. prolixus DH31 (ACX47068). 5. Bombyx mori DH31 (NP_001124379). 6. Apis mellifera DH31 (P85830). 7. N. vitripennis DH31 (XP_001599948). 8. T. castaneum DH31 (EEZ99367). 9. D. punctata DH31 (P82372). 10. Acyrthosiphon pisum DH31 (XP_001945901). 11. hCT (AAA58403). 12. hCGRP (1005250A). 13. R. norvegicus CT (AAA40849). 14. R. norvegicus CGRP (NP_612522). 15. G. gallus CT (ABY65359) 16. G. gallus CGRP (P10286). 17. Samo salar CT (NP_001135058). 18. S. salar CGRP (NP_001140052). Predicted hormone sequences: 2–3, 6–8 and 10; translated from cloned cDNAs: 1, 4–5, 9, 11–18. (C) Predicted protein structure of the Aaeg-DH31. Amino acid residues in pink are conserved in human CT. (PDF) [file pone.0050374.s004.pdf]

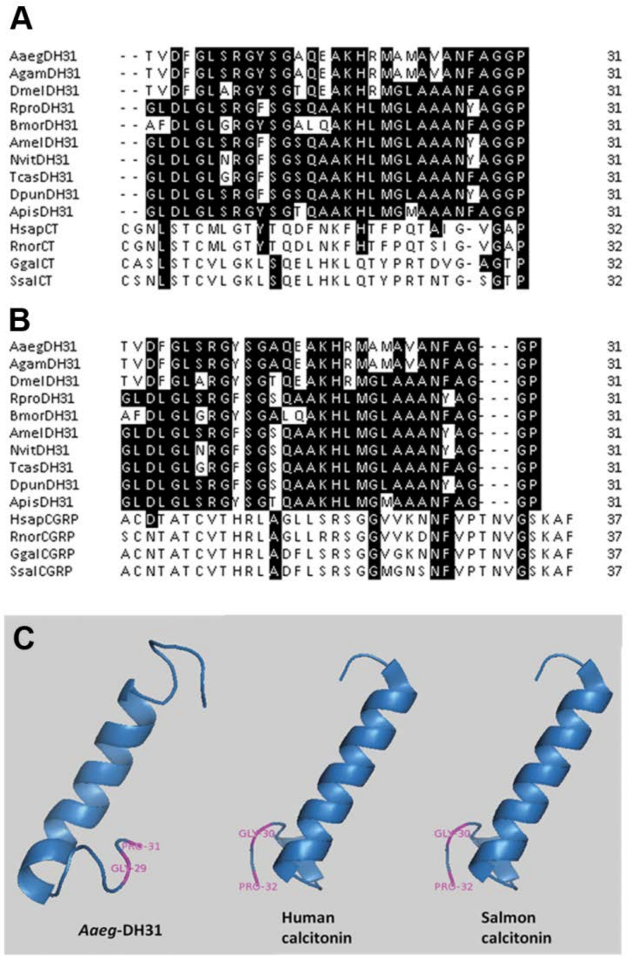

Figure S4. Amino acid sequence alignment of calcitonins (CTs) and calcitonin gene related peptides (CGRPs). (**A**) The *A. aegypti* diuretic hormone 31 (*Aaeg*-DH_{31}) sequence was aligned with those of other arthropod DH_{31} and CTs and in (**B**) with CGRPs. In (**A**) and (**B**), GenBank accession numbers: 1. *A. aegypti* DH_{31} (EAT40182). 2. *A. gambiae* DH_{31} (XP_321755). 3. *D. melanogaster* (AAF52685). 4. *R. prolixus* DH_{31} (ACX47068). 5. *Bombyx mori* DH_{31} (NP_001124379). 6. *Apis mellifera* DH_{31} (P85830). 7. *N. vitripennis* DH_{31} (XP_001599948). 8. *T. castaneum* DH_{31} (EEZ99367). 9. *D. punctata* DH_{31} (P82372). 10. *Acyrthosiphon pisum* DH_{31} (XP_001945901). 11. hCT (AAA58403). 12. hCGRP (1005250A). 13. *R. norvegicus* CT (AAA40849). 14. *R. norvegicus* CGRP (NP_612522). 15. *G. gallus* CT (ABY65359) 16. *G. gallus* CGRP (P10286). 17. *Samo salar* CT (NP_001135058). 18. *S. salar* CGRP (NP_001140052). Predicted hormone sequences: 2-3, 6-8 and 10; translated from cloned cDNAs: 1, 4-5, 9, 11-18. (**C**) Predicted protein structure of the *Aaeg*-DH_{31}. Amino acid residues in pink are conserved in human CT.
