# Supplementary material for: Role in Diuresis of a Calcitonin Receptor (GPRCAL1) Expressed in a Distal-Proximal Gradient in Renal Organs of the Mosquito Aedes aegypti (L.)
Source: PLoS One. 2012 Nov 29;7(11):e50374. doi: 10.1371/journal.pone.0050374 (PMC3510207; doi:10.1371/journal.pone.0050374)
Supplement: Figure S5 — Immunolocalization of Aaeg GPRCAL1 along the length of a single MT. The receptor signal (red, white arrows) was observed in only particular principal cells, the majority located towards the distal end which contains the tip cell. (PDF) [file pone.0050374.s005.pdf]

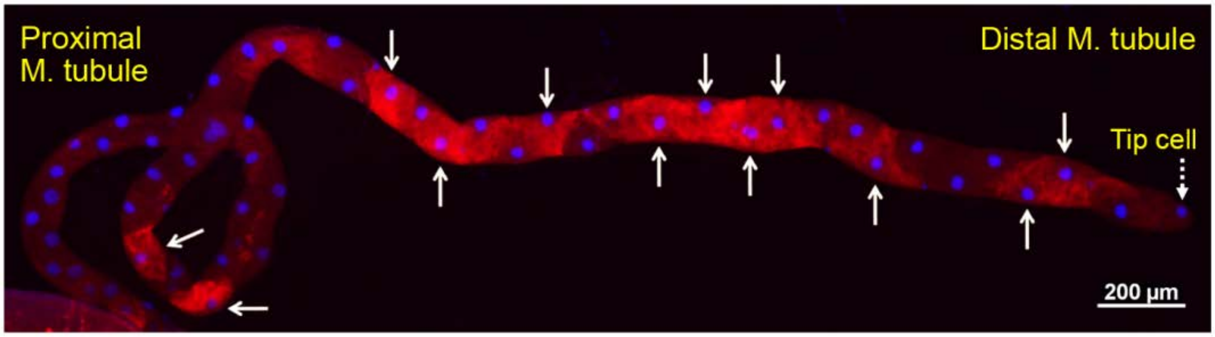

Figure S5. Immunolocalization of *Aaeg*GPRCAL1 along the length of a single MT. The receptor signal (red, white arrows) was observed in only particular principal cells, the majority located towards the distal end which contains the tip cell.
