# Supplementary material for: Role in Diuresis of a Calcitonin Receptor (GPRCAL1) Expressed in a Distal-Proximal Gradient in Renal Organs of the Mosquito Aedes aegypti (L.)
Source: PLoS One. 2012 Nov 29;7(11):e50374. doi: 10.1371/journal.pone.0050374 (PMC3510207; doi:10.1371/journal.pone.0050374)
Supplement: Figure S6 — Localization of Aaeg GPRCAL1 in MTs from females injected with AaegGPRcal1 dsRNA, EGFP dsRNA and water. (A) MTs from AaegGPRcal1 knock-down females exhibited reduction of receptor signal intensity (in this image by a factor of 2) compared to those of controls (B) EGFP dsRNA and (C) water. (PDF) [file pone.0050374.s006.pdf]

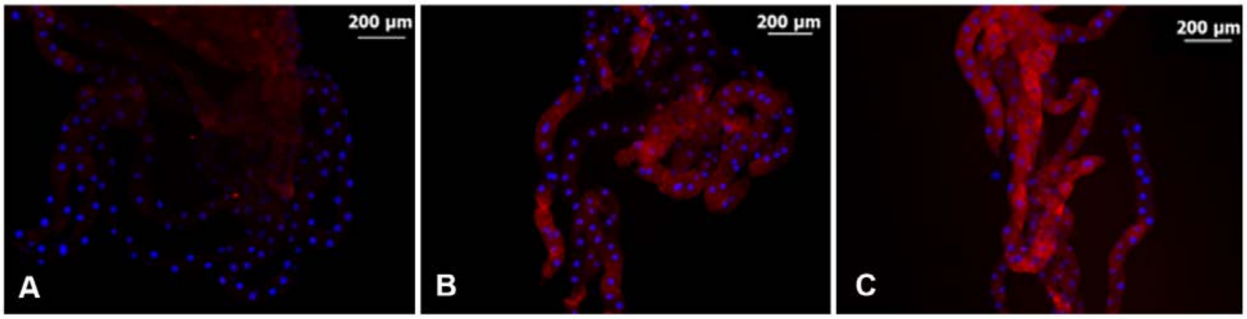

Figure S6. Localization of *Aaeg*GPRCAL1 in MTs from females injected with *AaegGPRcal1* dsRNA, EGFP dsRNA and water. (**A**) MTs from *AaegGPRcal1* knock-down females exhibited reduction of receptor signal intensity (in this image by a factor of 2) compared to those of controls (**B**) EGFP dsRNA and (**C**) water.
