# Supplementary material for: Role in Diuresis of a Calcitonin Receptor (GPRCAL1) Expressed in a Distal-Proximal Gradient in Renal Organs of the Mosquito Aedes aegypti (L.)
Source: PLoS One. 2012 Nov 29;7(11):e50374. doi: 10.1371/journal.pone.0050374 (PMC3510207; doi:10.1371/journal.pone.0050374)
Supplement: Table S1 — Primers used for cloning, transcriptional and functional analyses (RNAi) of AaegGPRcal1. (PDF) [file pone.0050374.s007.pdf]

Table S1. Primers used for cloning, transcriptional and functional analyses (RNAi) of *AaegGPRcal1*.

**Primers for PCR**	Sequence (5’-3’)	Orientation	cDNA location number /amino acid in Fig. S1
AADH31R7605	TTCCACCGGTCACAATAGTAAGACTAAGC : 29 bp	Antisense	1715-1743/ 3’UTR
AADH31F29406	TGCCAGATATCAACCAAACACACAAACCCAG : 31 bp	Sense	-88 to -58/5’UTR
AADH31F37609	ATGGTTGGCTCTGTTGGCCGGATACTGCG : 29 bp	Sense	134-162/ DGWLCWPDTA
AADH31R37799	TTCTCCGTCCTCATCGCAGTCTTTGTGTGCG : 31 bp	Antisense	225-225/FAHKDCDEGE
AADH31F65768	GCTCGCATTACGCTACATATGAACCTGTTTGCG : 33 bp	Sense	433-465/ARITLHMNLFA
AADH31F68248	ATCGAGAACCATACTGCAAGCGTTCAGAGCC : 31 bp	Sense	921-951/PSRTILQAFRA
AADH31R68434	GGGTCGGAACGGAGTCAATATGTACTGTAGGCC : 33 bp	Antisense	976-108/GLQYILTPFRP
**Primers for quantitative PCR**			
AADH31FQPCR3’ORF	TGGCGCACCGTGTTCTT : 17 bp	Sense	1138-1154/WRTVFL
AADH31RQPCR3’ORF	GACACTTGCGTTGCGGTGTA : 20 bp	Antisense	1175-1194/YTATQVS
P178 (β-actin)	GACTACCTGATGAAGATCCTGAC : 23 bp	Sense	*A. aegypti* actin ((Aaeact-1)
P179 (β-actin)	GCACAGCTTCTCCTTAATGTCAC : 23 bp	Antisense	Accession number : U20287
**Primer for synthesis of dsRNA**			
AADH31FT75’UTR	TAATACGACTCACTATAGGGATTCTTTGCCAGATATC AACCAAACACAAACCC : 53 bp	Sense	-94 to -60/5’UTR
AADH31RT75’ORF	TAATACGACTCACTATAGGGTTCTCCGTCCTCATGCC AGTCTTTGTGTGCG : 51 bp	Antisense	270-300/FAHKDCDEDGE
T7P164 (EGFP)	TAATACGACTCACTATAGGGACGTAAACGGCCACAA GTTCAGCGTGTC: 48 bp	Sense	
T7P165 (EGFP)	TAATACGACTCACTATAGGGTCACGAACTCCAGCAG GACCATGTGATC : 48 bp	Antisense	

Numbers assigned for primers for PCR correspond to the first 5’ nucleotide location in contigs AAGE02017873 or AAGE02019029
